# Continuity in morphological disparity in tropical reef fishes across evolutionary scales

**DOI:** 10.1038/s42003-025-07634-7

**Published:** 2025-02-17

**Authors:** Giulia Francesca Azzurra Donati, Camille Albouy, Thomas Claverie, David Mouillot, Rodney Govinden, Oskar Hagen, Shameel Ibrahim, Julius Pagu, Irthisham Zareer, Fabien Leprieur, Loïc Pellissier

**Affiliations:** 1 Ecosystems and Landscape Evolution, Institute of Terrestrial Ecosystems, ETH Zürich, CH-8092 Zürich, Switzerland; 2 Swiss Federal Research Institute WSL, CH-8903 Birmensdorf, Switzerland; 3 Swiss Federal Institute of Aquatic Science & Technology (Eawag), Überlandstrasse 133, Dübendorf, Switzerland; 4 UMR ENTROPIE (UR-IRD-CNRS), Université de La Réunion, Faculté des Sciences et Technologies, La Réunion, France; 5 MARBEC, Univ Montpellier, CNRS, IFREMER, IRD, Montpellier, France; 6 Institut Universitaire de France, Paris, France; 7 Seychelles Fishing Authority, Mahe, Seychelles; 8 German Centre for Integrative Biodiversity Research (iDiv) Halle-Jena-Leipzig, Leipzig, Germany; 9 Maldives Whale Shark Research Programme, Popeshead Court Offices, Peter Lane, York, Yorkshire UK; 10 Mafia Island Marine Park, Mafia, Tanzania

**Keywords:** Evolutionary theory, Evolutionary ecology

## Abstract

Tropical reef fishes exhibit a large disparity of organismal morphologies contributing to their astonishing biodiversity. Morphological disparity, scaling from differences among individuals within populations to differences among species, is governed by ecological and evolutionary processes. Here, we examined the relationship between intra- and interspecific disparity in 1111 individuals from 17 tropical reef fish species, representing 10 families with different dispersal abilities, across four Indian Ocean regions. We compared intraspecific measurements with species-level measures from a database of 1061 reef fish species. Species with high morphological disparity among individuals from distinct regions are found to be nested in families that display a high disparity among their genera. We show an association between the morphological disparity at the intra- and interspecific levels for several morphological ratios such as the caudal peduncle elongation. We evaluated the link between morphological disparity and genetic diversity with species dispersal ability. A structural equation model indicates that dispersal ability correlates positively with species genetic diversity, which is associated with morphological disparity. Our results suggest that traits associated with dispersal may foster gene flow and morphological evolution. Future works combining genomic, morphological and environmental data across more species is necessary to generalize these findings to other regions.

## Abstract (web summary)

Morphological disparity in tropical reef fishes links intra- and interspecific variation, shaped by ecological and evolutionary processes. Dispersal traits promote genetic diversity and disparity, highlighting their role in gene flow and morphological evolution.

## Introduction

Animals display spectacular disparity in their morphology, prompting questions about how such variations emerge from ecological and evolutionary mechanisms^1,2^. The morphological disparity among individuals and species is partly derived from underpinned genetic diversity^3,4^, which in turn is determined by ecological^5^ and evolutionary processes^6^. Gene flow, modulated by dispersal, controls the overall gene pool of a species^7^ and potentially determines its adaptive capacity^7,8^. Evaluating the link between species’ dispersal ability and their capacity to express different phenotypes across geographically distant populations in distinct environments^9^ requires the combination of genetic, morphological and dispersal information in a regional interconnected system.

The micro- and macroevolution of species morphologies have been proposed to arise from corresponding processes^10^, where disparity emerging among individuals becomes fixed in deeper phylogenetic lineages^11^. In contrast, other studies have argued that micro- and macroevolutionary processes are decoupled because they happen at different time scales^12^. Studies comparing micro- to macroevolutionary changes in more biological systems could help better understand evolutionary scaling^13^. To disentangle phenotypic plasticity and adaptation, which act at different temporal scales but can promote intraspecific morphological disparity^14^, we must investigate the association between morphological variations measured within species (microevolution) and morphological variations measured in deeper phylogenetic lineages (macroevolution, e.g., genera within families^15^). The correspondence across evolutionary scales would support the heritability of morphological variations, while a lack of correspondence would suggest that such variations predominantly arise from plasticity or that other processes increase the complexity of the relationship (e.g., ref.^16^). Geographically segregated populations can display different morphologies, as shown for isolated sister species and this can reflect differences detected among deeper evolutionary lineages^17^. Hence, geographic sampling can be leveraged to quantify variation between individuals across distant locations to the variation observed at macro-evolutionary levels between species from the same genera.

Tropical reef fishes exhibit a wide range of dispersal abilities^18,19^ that can be influenced by the interplay between current dynamics and larval dispersal abilities, different levels of genetic diversity among species^20^, and large morphological disparities^21,22^, so could help to decipher the links between morphological disparity and genetic diversity among and within species. Coral reefs, known for their complex habitats and varied hydrologic conditions at the interface between benthic and pelagic environments^23^, exert a variety of selective pressures on resident fish species. These pressures can lead to significant morphological adaptations as species optimize their body shape to thrive in distinct reef environments^24^. Therefore, coral reef habitats are prone to promote the evolution of morphological disparity within associated fish families^24^. This intraspecific morphological disparity has been shown to mirror the differences observed between species in some specific cases^25^, providing a unique opportunity to explore an evolutionary continuum from microevolutionary changes between geographically distant reefs to macroevolutionary changes between species. Understanding these patterns not only elucidates the mechanisms driving morphological diversity but also contributes to broader insights into the evolutionary processes shaping biodiversity in this very diverse ecosystem^23^.

**Fig. 1:**
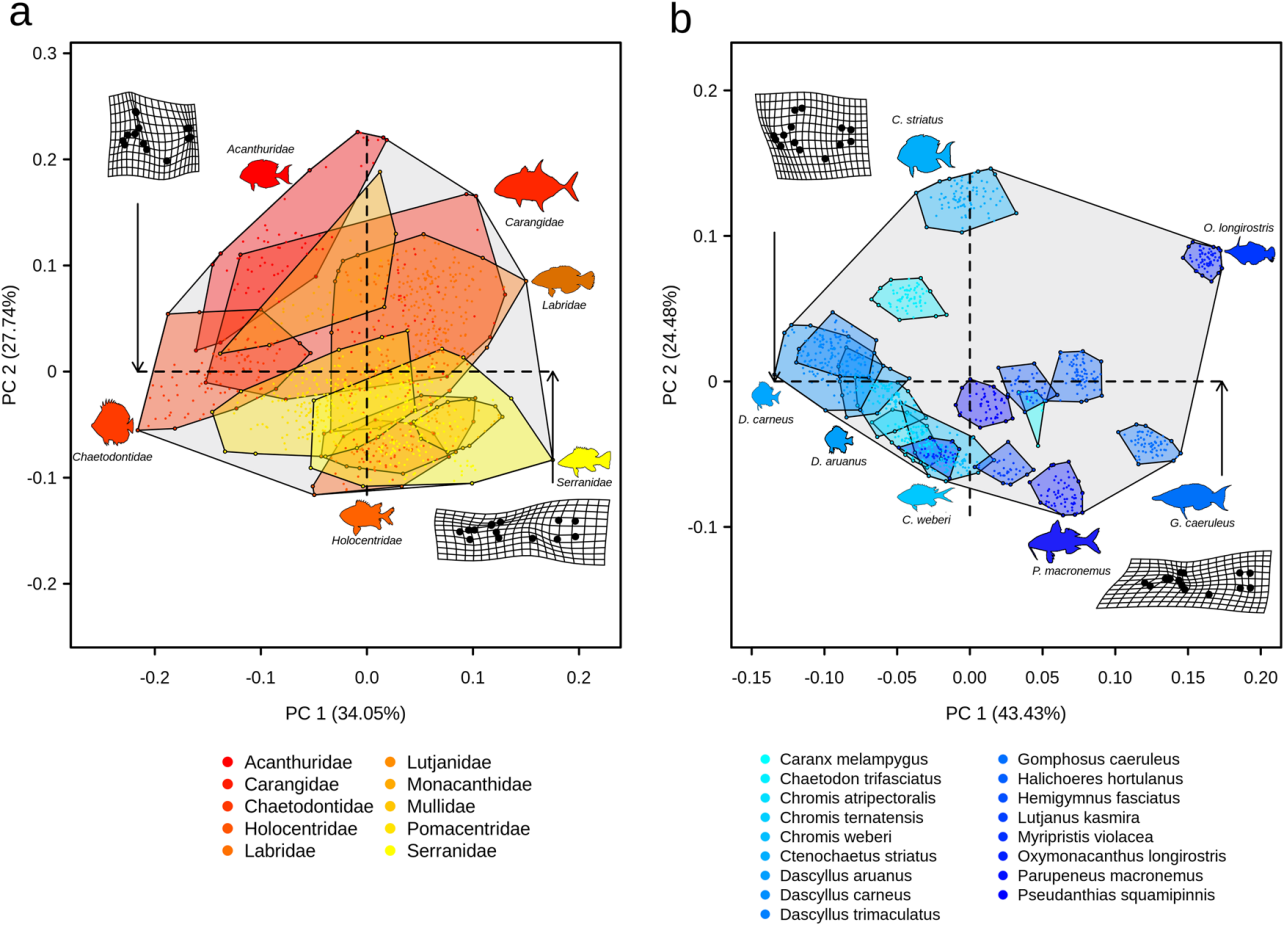
Morphospaces of both interspecific and intraspecific tropical reef fishes data sets. Morphospaces of the **a** interspecific and **b** intraspecific data sets of tropical reef fishes. Morphological spaces were obtained by performing principal component analyses (PCAs) on fish shapes characterized by 14 common body landmarks. **a** The morphological space of 1061 Indo-Pacific reef fish species, gathered from a subset of ^33^, **b** the morphological space of 1111 individuals belonging to 17 reef fish species sampled in 4 geographically distant locations across the Western Indian Ocean. Polygons represent a the spread of species in families and **b** the spread of individuals in species. The total amount of morphological space occupied by the considered tropical reef fishes and families, i.e., the morphological richness (MRic), is shown by the light grey polygons for the interspecific level (**a**, MRic = 0.09) and the intraspecific level (**b**, MRic = 0.049). The deformation grids illustrate the shape change at the extremes of the morphospace across **a** families and **b** species.

Here, we combined measurements of genetic diversity with data on body morphology at multiple geographic locations to test whether dispersal is associated with morphological disparity in tropical reef fishes at both intra- and interspecific levels. To do so, we selected 17 tropical reef fish species, from 10 common families, with varying adult body size and pelagic larval duration (PLD; Fig. 1 and Supplementary Tables 1 and 2), which are recognized as major dispersal traits in tropical reef fishes^26,27^. We sampled a total of 1111 individuals of these species in 4 geographically distant locations across the Western Indian Ocean (mean distance between pairwise locations: ca. 2200 km; Supplementary Table 2 and Supplementary Fig. 1a). We measured 13 morphological traits per individual based on distances between landmarks at specific locations on the fish body (Supplementary Table 3 and Supplementary Fig. 2). We used these ratios to compute morphological disparity between geographic locations (intraspecific level, see Methods, Eqs. 1–2), which we compared between the genera of the corresponding families based on interspecific level.

## Results and Discussion

### Dispersal and genetic diversity

Using a PCA based on adult body size and pelagic larval duration, we ranked the 17 species along a gradient of dispersal ability (Supplementary Fig. 3). The first PCA axis was strongly associated with body size (Pearson correlation test, *r* = 0.91, t = 8.2, DF = 15, *P* < 0.05) and PLD (Pearson correlation test, r = 0.91, t = 8.2, DF = 15, *P* < 0.05). We then related the species coordinates on the first PCA axis to genetic diversity, assessed using double-digest restriction-site-associated DNA sequencing (ddRAD-seq; Supplementary Table 4). We found a positive association with the regional genetic diversity (Ordinary Least Square regression; OLS: coeff = 0.017, R^2^ = 0.32, *P* = 0.018; Supplementary Fig. 3) and a negative association with the genetic differentiation among sample locations (OLS: coeff = -0.0013, R^2^ = 0.17 *P* = 0.01; Supplementary Fig. 3). This finding suggests that dispersal contributes to shaping the spatial genetic structure of tropical fish species^28–30^.

### Correspondence in intra- and interspecific morphological disparity

**Fig. 2:**
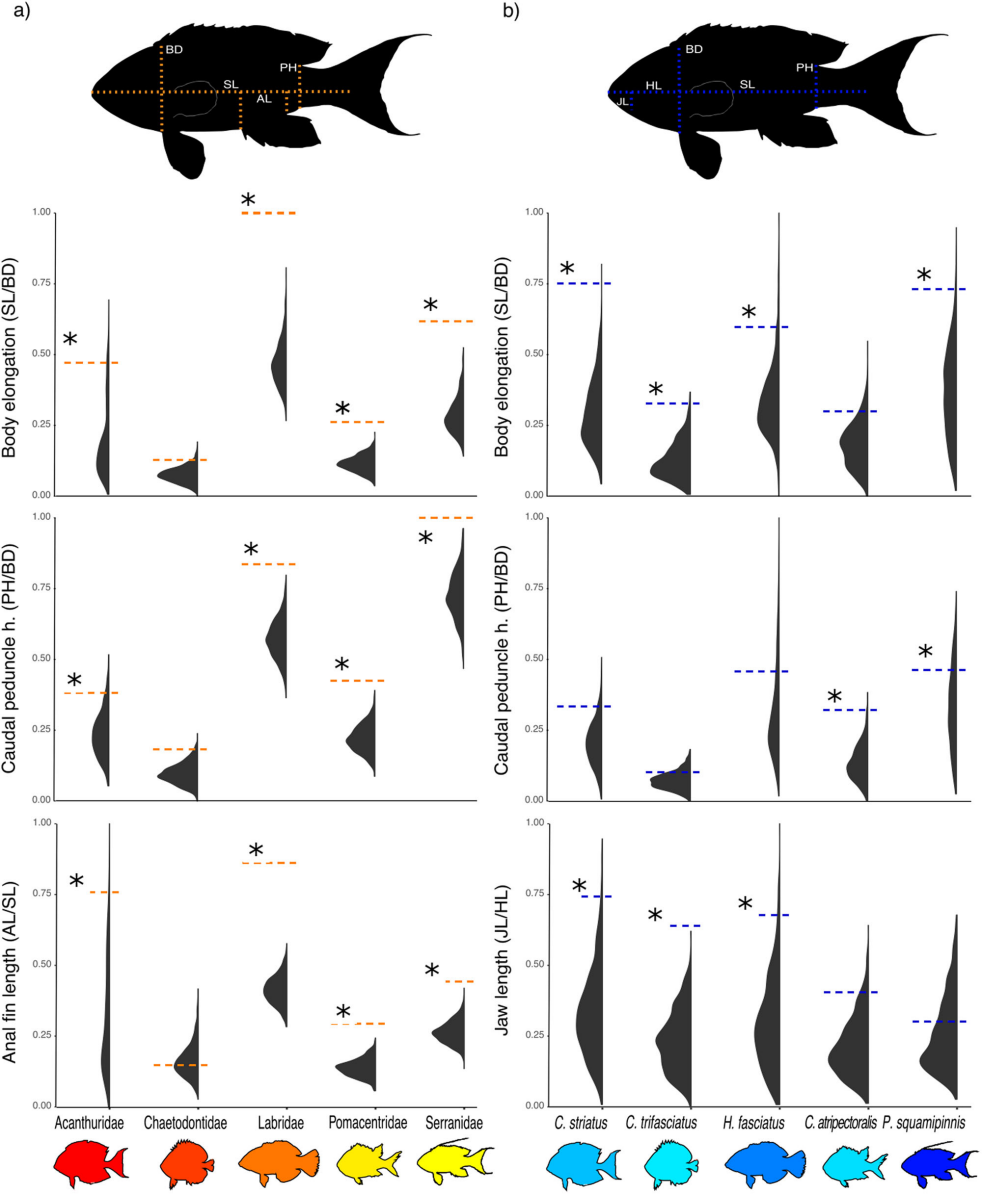
Morphological trait disparity in tropical reef fishes at both interspecific and intraspecific levels. Illustration of the morphological trait disparity in tropical reef fishes at the **a** interspecific and **b** intraspecific levels. The top panel represents axes of morphological disparity in tropical reef fishes. Colored dashed segments on fish shapes illustrate the relevant morphological trait, while the letter code indicates the morphological measure components of the morphological traits (BD body depth, SL standard length, AL anal fin length, PH caudal peduncle height, JL jaw length, HL head length). The bottom panels represent the observed values of body elongation, caudal peduncle and Anal fin length variabilities (colored dashed segments) and the distribution of morphological disparity obtained under null models (black shaded distributions: 999 randomizations; **a**: of the genus; **b**: of the population) for five families (**a**) and for five species of the same family (**b**), taken as an example (for more detail see Supplementary Figs. 4 and 5). The * indicates that the observed morphological disparity differs from that expected at random (SES > 1.96).

**Fig. 3:**
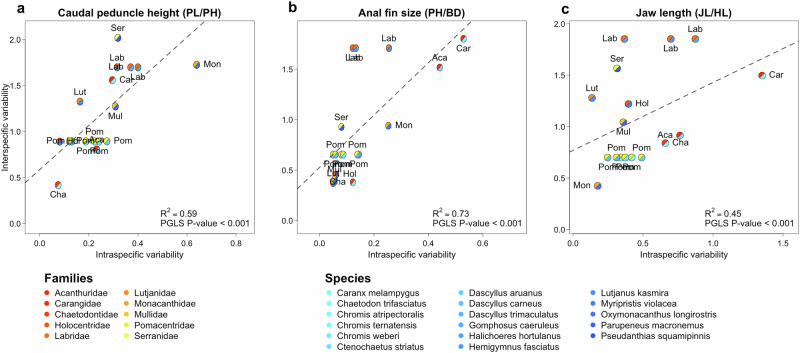
Relationships between the intraspecific and interspecific morphological disparity in tropical reef fishes. Relationships between intraspecific and interspecific variability for (**a**) caudal peduncle height (PL: caudal peduncle length, PH: caudal peduncle height); (**b**) anal fin (AL: anal fin length) and (**c**) Jaw-head length relationship (JL jaw length, HL head length). Each symbol contains two colors, one corresponding to the intraspecific data set and the other to the interspecific data set. The dashed line represents the Ordinary Least Square regression between intra- and interspecific morphological variability. The reported P-values in each panel correspond to the significance of the relation accounting for the phylogenetic relationship between species (PGLS phylogenetic generalized least-squares).

We compared intraspecific morphological disparity between geographic locations, based on 1111 individuals from 17 species (Fig. 1b) to interspecific disparity using a morphometric data set that includes 1,061 Indo-Pacific reef fish species. (Fig. 1a, Supplementary Table 5). We used a co-inertia analysis based on two separate principal coordinates analyses (PCoA) and showed a general correspondence between the morphological disparity at the intra- and interspecific levels (RV coefficient 0.44; COIA, *P* = 0.02, rep = 99). Species that presented higher morphological disparity among geographic locations than expected by a null model (Fig. 2 and Supplementary Figs. 4–5) were also those with elevated disparity within their family (e.g., Labridae, Acanthuridae). For each morphological trait, we also related the intraspecific morphological disparity among locations to the disparity among genera within their respective families using both ordinary Least squares (OLS) and phylogenetic generalized least squares (PGLS) models. We found that individual relationships were significant for 6 out of the 13 morphological traits after having controlled for phylogenetic non-independence in our data set, namely caudal peduncle elongation, caudal-peduncle body height, jaw-head length, standardised anal fin length, standardised pre-anal length and body elongation (Fig. 3, Supplementary Table 3 and Supplementary Fig. 6). These relationships were found to be particularly marked for the caudal peduncle body height (OLS: R^2^ = 0.59; PGLS: coeff = 0.29, *P* < 0.001; Fig. 3), the standardized anal fin length (OLS: R^2^ = 0.73; PGLS: coeff = 0.28, *P* < 0.001) and the jaw-head length (OLS: R^2^ = 0.45; PGLS: coeff = 0.53, *P* < 0.001, Fig. 3, see Supplementary Fig. 6 for all traits considered). Those relationships were robust for 5 out of the 13 morphological traits after the removal of *Caranx melampygus* which displays the largest body size and greatest dispersal ability in our data set (see Supplementary Figs. 7-8). However, it is worth noting that the removal of this species strongly reduced the % of explained variance (R^2^) in the OLS models for body elongation (OLS R^2^ = 0.62 and 0.25, for the models with and without *Caranx melampygus*, respectively, see Supplementary Figs. 6a and 8a). Overall, our analyses suggest a correspondence of morphological disparity, from within species to within families for certain traits (e.g., caudal peduncle length, caudal peduncle height anal fin length) and call for further exploration including a broader spectrum of species, particularly large-bodied species with a high dispersal ability.

### Association between genetic diversity and morphological disparity

**Fig. 4:**
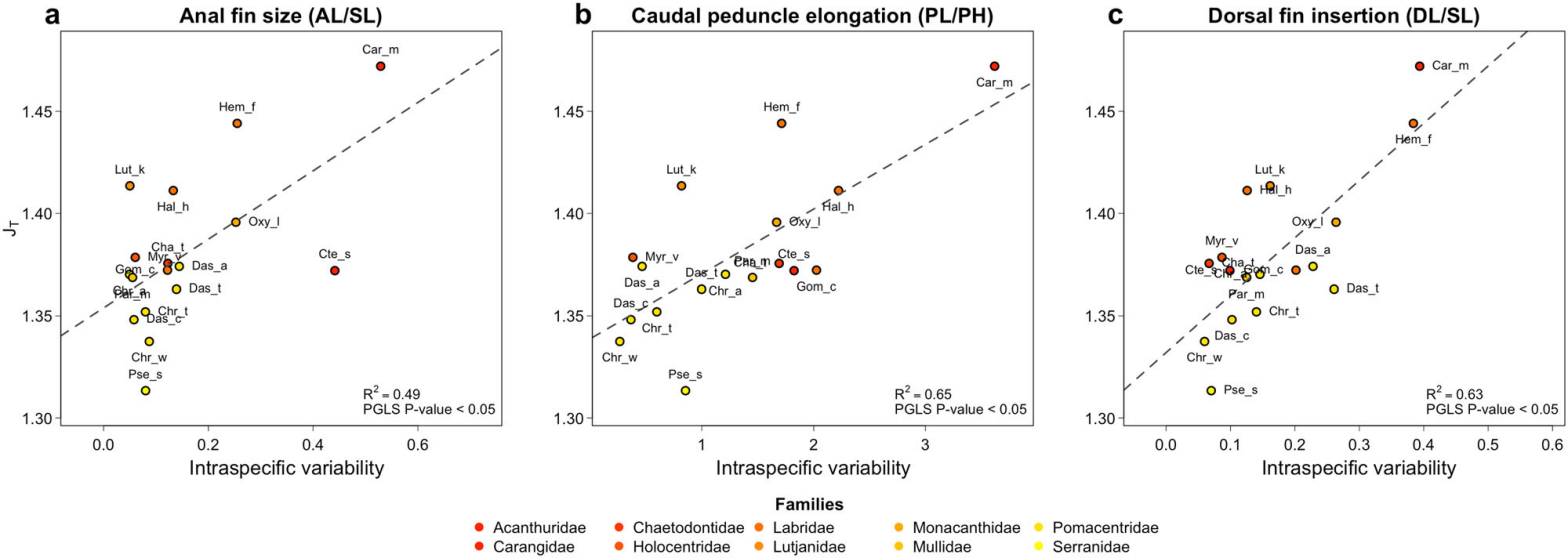
Relationships between the total intraspecific genetic diversity (J_T_) and the morphological trait variation for 17 tropical reef fish species. Phylogenetic generalized least squares (PGLS) relationships between total intraspecific genetic diversity of the Western Indian Ocean and the variability in (**a**) relative anal fin size (AL anal fin length, SL standard length) (**b**) caudal peduncle elongation (PL caudal peduncle length, PH caudal peduncle height), and (**c**) the dorsal fin insertion (DL dorsal fin length) at the intraspecific level. The dotted line represents the Ordinary Least Square regression between total genetic diversity (J_T_) and intraspecific morphological disparity. The reported P-values in each panel correspond to the significance of the relation accounting for the phylogenetic relationship between species (PGLS phylogenetic generalized least-squares).

To quantify the different components of genetic diversity (J_T_, total genetic diversity; J_S_, mean within-population genetic component and J_ST_ the between-population component) and relate them to morphological disparity we applied a multiplicative partitioning framework based on Hill’s number (J_T_ = J_S_ x J_ST_^31^; Supplementary Table 6). The total genetic diversity (J_T_) of each reef fish species considered in this analysis, and not the variation (J_ST_), was significantly associated with intraspecific morphological disparity (Table 1; PERMANOVA J_T_: R^2^ = 0.21; *P* = 0.007) for several morphological traits. The relationships between genetic diversity and morphological disparity were significant for 9 out of the 13 morphological traits considered (caudal peduncle elongation, standardised anal fin length, eye-head size relationship, jaw-head length relationship, body elongation, standardised pre-dorsal fin length, eye-head position relationship, standardised Pre-anal length, standardised dorsal fin length; (Fig. 4 and Supplementary Figs. 9; Supplementary Table 3), and for 7 out of 13 when removing the Carangidae family (Supplementary Figs.  10-11). Associations between genetic diversity and morphological disparity were particularly marked for morphometric features related to the fish head region, with variations in the relative position and size of the eye, in the relative jaw length and relative head length (Supplementary Fig. 2), and in features related to fins (Supplementary Fig. 2). A synthetical structural equation model (SEM) supported positive relationships between dispersal capacity, total genetic diversity, and morphological disparity within species (SEM: PCA axis1 vs. γ diversity, Std.estimate = 0.66, DF = 14, *P* = 0.0019; PCoA_intra vs. γ diversity, Std.estimate = 0.74, DF = 14, *P* = 0.0037; Supplementary Fig. 12). The positive causal path between species dispersal capacity, regional genetic diversity, and morphological disparity indicates an association between dispersal, genetic diversity and adaptation. In contrast, we found a negative relationship between dispersal and genetic differentiation (SEM: PCA axis1 vs. *β* diversity, Std.estimate = -0.53, DF = 14, *P* < 0.05; Supplementary Fig. 12).

**Table 1:** Linking intraspecific morphological disparity to genetic diversity

		DF	SS	R^2^	*F*	*P*
Genetic diversity	J_T_	1	0.21	0.21	4.08	0.007*
	J_ST_	1	0.065	0.0652	1.190	0.289
	Residuals	14	0.732	0.727		
	Total	16	1.008	1		

Permutational analysis of variance (PERMANOVA) performed between the 13 morphological traits used to compute intraspecific morphological disparity between geographic locations and the genetic diversity descriptors obtained by applying a Hill number framework on genome-wide SNP data (see methods for details).

For the 17 species of the intraspecific data set (Supplementary Table 1), tests were run using Euclidean distances among samples and 999 permutations. The model terms were added sequentially: total genetic diversity (J_T_) followed by genetic differentiation (J_ST_). Degrees of freedom (DF), sum of squares (SS), and significance (*P*) are reported. Significant effects (*P* < 0.05) are indicated with asterisks.

### Morphological disparity and associated functions

Fish morphological traits are closely tied to their biological functions^23,31^. Fin features enhance speed and agility, aiding in predator evasion and efficient foraging^32^, while mouth and head shapes and sizes reflect feeding strategies^33^. We found that morphological traits associated with the fish head and fins showed correspondence from intra- to interspecific, typically associated with nutrition and movement^34,35^. Longer jaw features relative to their bodies are often adapted for biting or capturing larger prey^36,37^, while smaller jaws are suited for grazing or filter feeding. Similarly, fin size and shape affect swimming efficiency^35,38^ with larger fins aiding in stability and maneuverability in complexly structured environments, and more streamlined fins supporting fast, sustained swimming in open water. Therefore, changes in shape along these axes are thought to have implications for swimming performance, defense from gape-limited predators, suction feeding performance and access to specific habitats. Our results agree with the significant increase in morphological disparity in species with larger body size documented by^39,40^ and support the heritability of the measured variations for the traits that showed significant congruence in morphological disparity across evolutionary scales^10^.

### Dispersal, genetic diversity and morphological disparity

Dispersal modulates the level of gene flow which determines population genetics, their degree of local adaptation^41^ and speciation^42^. We found that dispersal was positively associated with both genetic γ diversity and morphological diversity in tropical reef fishes. Theory predicts that dispersal can influence distinct adaptation among populations, both positively^43^ and negatively^8,44,45^, depending on the frequency at which non-neutral genetic differentiations arise^46^. When genetic differentiations arise locally at sufficient frequency, divergent environmental selection can promote morphological disparity among populations in the absence of any other driver^47^. In that case, dispersal tends to homogenize the phenotypic disparity among geographic groups^44^. In contrast, when genetic differentiations arise only rarely, dispersal can increase the exchange of genetic variations between geographically isolated populations, which can fuel selection and phenotypic differentiation^46^. Our results suggest that morphological disparity emerges faster in species with higher dispersal and genetic exchanges. This process may allow selection to act on a broader range of genetic material to shape intraspecific morphological disparity among locations. In contrast, we found a negative relationship between dispersal and genetic differentiation meaning that low dispersal ability is likely to limit gene flow between geographic groups, eventually generating new species through allopatric speciation^48,49^. Limited dispersal can attenuate gene flow and promote population structure and ultimately allopatric speciation^19^. Species with the most limited dispersal abilities and most distinct genetic structure typically belonged to the most species-rich families, such as Pomacentridae and Serranidae. While our findings agree with a decline in speciation rate as dispersal ability increases^48,49^, the positive correlation between dispersal ability and morphological disparity suggests that the process determining the number of species neutrally, (e.g., via Dobzhansky-Muller incompatibilities) differs from the process that shapes morphological disparity among lineages.

### Limitations and future perspectives

The observed correspondence between intra- and interspecific morphological disparity suggests that the expression of morphological disparity may be heritable and thus would partly arise from local adaptation through natural selection^50^ rather than from environmentally induced plasticity^14^. However, we cannot refute that neutral processes and phenotypic plasticity also contribute to morphological disparity in tropical reef fishes^51^. In addition, the neutral markers from Rad-seq that we used are not a direct measurement of adapted loci^52^ and genetic diversity may not reflect adaptive potential. It is also worth noting that the collection of intraspecific data for tropical fishes across multiple locations and species requires a significant effort and our analyses are limited by the number of species considered with only 17 species across 10 families. Future studies should aim to take similar measurements across more individuals, species and sites. In addition, the selection of species was partly determined by their relative abundance on the sampled reefs, which means that our set of selected species is not evenly distributed across reef fish families and lacks representation of species from the larger size spectrum. Because some families were over-represented with more than one species (i.e., Labridae and Pomacentridae), we used phylogenetic regression to account for shared ancestry, as well as an averaging approach based on their taxonomic classification (Supplementary Fig. 13), which did not affect our main conclusion. Future work combining genomic, morphological and environmental data over a large spatial scale for multiple species could help to provide a mechanistic link between gene expression pathways, morphological disparity and speciation^53^.

## Conclusion

Morphological disparity among species emerges from processes happening within and among populations, as evidenced by correspondence across evolutionary scales. However, it remains to be explored whether the relationships documented here in the Western Indian Ocean are confirmed with more species and in other regions to test their generality. We further demonstrated associations between dispersal, genetic diversity and morphological disparity. Connectivity among populations is key to avoiding inbreeding depression^54^, and our results suggest that dispersal might increase rates of adaptation across different habitats. Given the unprecedented rate of changes in marine environmental conditions associated with increasing anthropogenic pressure^55^, maintaining the connectivity between populations, especially across marine protected areas^56^, is essential to foster the exchange of genetic materials to facilitate adaptation.

## Methods

### Sampling design and species selection

Before sampling, 17 target species were selected, through a multidimensional ecological trait analysis^57^, ranging from small- to large-bodied and short to long PLDs, and including various abundances on the reef, to ensure that the species were representative of the tropical reef fish dispersal ability in the Western Indian Ocean (Supplementary Table 1). To perform this species selection we ran a principal coordinate analysis (PCoA) over all species co-occurring in the target sampling locations (*n* = 2292) using five ecological traits gathered from Fishbase^58^ and the literature, namely: (*i*) adult body size (cm); (*ii*) PLD (days); (*iii*) adult home range mobility (narrow versus wide); (*iv*) reproductive guild (pelagic versus benthic spawners); and (*v*) schooling (groups with less than 20 versus greater than 20 individuals). For the selected species, we considered an estimation of their regional scale species abundance as a proxy of population census size^59^ (i.e., the cumulative number of individuals per transects over the WIO, gathered from the Reef Life Survey program, https://reeflifesurvey.com).

Following this selection, a total of 1111 individuals belonging to these 17 tropical reef fish species were sampled from populations near the Maldives (central atolls), Mafia Island (Tanzania), Mayotte Island (Comoros Archipelago), and Seychelles (Supplementary Table 2, Supplementary Fig. 1). Sampling was conducted using hand barrier nets while scuba diving, in compliance with local regulations. For the largest-bodied target species (e.g., *Caranx melampygus*), were additionally collected from local fish markets, where the fishing location of the fish was known^31^.

### DNA extraction and genotyping

Muscle tissues were sampled from the individuals of the 17 target species collected from all four Western Indian Ocean geographically isolated locations^60^ (i.e., Maldives, Mayotte Island, Mafia Island and Seychelles; Supplementary Fig. 1). High-quality genomic DNA was extracted from the muscle tissue using the sbeadex livestock kit (LGC Biosearch Technologies, Teddington, UK; catalog numbers 44701 and 44702). ddRAD-seq libraries were prepared using EcoRI and Taq1a (New England Biolabs, Inc., Ipswich, MA, USA) following the protocol used in^61^, which is a modified version of the procedure used in ref. ^62^. In total, 24 ddRAD-seq library pools containing 2 × 48 internal barcodes each were sequenced in 12 lanes on the Hiseq 2500 Illumina platform using the 2 × 125 bp protocol (Fasteris, Geneva, Switzerland). The default settings of the dDocent pipeline v.2.2.25^63^ were used to obtain the genotypes. Briefly, raw reads were demultiplexed using Stacks (v2.0b)^64^. A reference catalogue was built de novo for each species. To find the optimal parameters, the remapping rate was maximized by varying the coverage of unique sequences within individuals, the number of shared loci among samples, and the sequence identity (%)^31^. The reads were remapped to the reference catalogs using BWA v.0.7.17^65^ and SNPs were called using FreeBayes v.1.3^66^. As recommended in ref. 67, total SNPs were filtered using VCFtools v.0.1.16 and vcflib v.1.0.1^68^ to retain only high-quality SNPs. Only variants (SNPs) that had been successfully genotyped with a minimum quality score of 20, minimum mean depth of 3, mean depth of 10, minor allele count of 3, and minor allele frequency of 5% were retained. Additionally, loci with > 20% missing data per population were removed. Filtering for allele balance and mapping quality between the two alleles was carried out, loci with coverage that was too high were removed, complex SNPs were decomposed into single SNPs, indels and sites with missing data ( > 5%) were removed, and only biallelic sites in Hardy-Weinberg equilibrium were kept. Finally, a RAD (restriction site associated DNA) haplotyper^69^ was used with the default settings to remove putative paralogous loci. Differences in population sampling success were accounted for by standardizing the sample size to a maximum of 10 individuals per population (median and tradeoff value of the overall sampling). Additionally, the filtered SNP data were down-sampled 99 times to the lowest common number of SNPs (i.e., 4479) found across all species (Supplementary Table 4).

### Experimental photographic design

Each specimen was pinned head facing left on a board to have the most accurate view of the full body shape including fin extensions. To overcome occasional post-mortem rigidity deformations, the epaxial muscle was massaged. Each specimen was photographed together with a scale bar using a digital-single-lens-reflex camera (24.2 megapixels, D7200, Nikon, Tokyo, Japan). An AF Micro-NIKKOR 60 mm F/2.8D lens (Nikon) was used to avoid distortion. The geometric morphometric data were collected for 1111 individuals (17 species, 10 families, Supplementary Table 2), forming the “intraspecific” data set. The resulting images were first imported into tpsUtil32^70^. Next, a collection of discrete anatomical landmarks described by two-dimensional cartesian coordinates (morphological landmarks), were digitized by one observer, using tpsDIG v.3.2^70^ to capture significant body-shape feature measurements related to fish feeding and locomotion mechanics (Supplementary Fig. 2). A subset of overlapping landmarks (*n* = 14) was gathered from^33^ and described 1061 Indo-Pacific reef fish species from the same families. Based on these landmarks a morphospace representation was done through relative warp analysis. This “interspecific” data set included one adult individual per species, with measurements made on photographs from Dr. Jack Randall (Bishop Museum, Honolulu, Hawaii, USA; http://pbs.bishopmuseum.org/images/JER/images.asp). Each landmark data set was first aligned using Generalized Procrustes Analysis (GPA), which rotates, translates and scales landmark configurations^71^. GPA makes it possible to isolate shape information, removing all other components of variation^34^. All subsequent analyses were conducted on the data sets with procrustes-aligned landmark coordinates (shape data).

### Intraspecific and interspecific morphological trait disparity

From the procrustes-aligned landmark data sets, two data sets were created representing the intra- and interspecific morphological trait disparity (mtD) for the 13 morphological traits (Supplementary Fig. 2; Supplementary Table 3). First, in both data sets, the morphological trait lengths were extracted from the Euclidean distances between landmarks. Second, the linear distances were converted into ratios to describe components of body and head shape (Supplementary Table 3). To compute the intraspecific morphological trait disparity (mtD), the disparity of traits per species across all populations in the Western Indian Ocean was considered, while the disparity of traits across all genera constituting a given family was considered in calculating the interspecific mtD. mtD was computed using Eq. 1:

1 $${mtD}=\frac{{\sum}_{i}{\sum}_{j}\left|{\bar{x}}_{i}-{\bar{x}}_{j}\right|}{{\bar{x}}_{{all}}}$$

where ${\bar{x} }$ stands for the mean value of a morphological trait (e.g., body elongation). For the intraspecific data set ${\bar{x}}_{i}$ refers to the mean of a morphological trait for location *i* (e.g., Maldives *and* ${\bar{x}}_{j}$ refers to the mean of a morphological trait for another location *j* (e.g., Mayotte island), while for the interspecific data set ${\bar{x}}_{j}$ refers to the mean of a morphological trait for genera *i*. ${\bar{x}}_{{all}}$ represents the mean value relative to the same morphological trait (e.g., body elongation) considered over the both location together for the intraspecific data set (i.e., mean of the mean of the four populations) and over the family for the intraspecific data set (i.e., mean of the mean of all genera in a family). ${\bar{x}}_{{all}}$ was calculated according to Eq. 2:

2 $${\bar{x}}_{{all}}={\sum }_{i=1}^{N}{\bar{x} }_{i}* \frac{1}{N}$$

We applied a null model approach to test whether interspecific mtD differed from that expected at random, we first calculated the observed trait variability between separated locations of the Western Indian Ocean using Eq. 1. Second, we randomized individuals between locations to obtain a distribution of 999 values of intraspecific trait variations. We then calculated the standard effect size (SES) to reject the null hypothesis (H0: the interspecific mtD does not differ from expected at random) or not. Similarly, to test whether intraspecific mtD differed from that expected at random, we first calculated the observed trait variability between genera for a considered family using Eq. 1. Second, we randomized species between genera of a family to obtain a distribution of 999 values of interspecific trait variations. We then calculated the SES to reject H0 (H0: the intraspecific mtD does not differ from expected at random) or not. The statistical framework was applied to each morphological trait ratio (*n* = 13).

### Linking genetic diversity in the Western Indian Ocean metapopulation to within-species morphological trait disparity

The *α, β* and *γ* components of genetic diversity were quantified by applying a multiplicative partitioning framework for genetic diversity based on Hill’s number^31^, expressed as ${J}_{T}={J}_{S}\times {J}_{{ST}}$, where *J*_*T*_ represents the overall genetic diversity. *J*_*S*_ represents the mean within-population genetic component $(\underline{\alpha })$ and is expressed as ${J}_{S}=1/\left(1-{H}_{S}\right)$, where *H*_*S*_ is the heterozygosity of populations^72^. *J*_*ST*_, expressed as ${J}_{{ST}}=1/\left(1-{H}_{{ST}}\right)$, represents the between-population (*β*) component, where ${H}_{{ST}}=\left({H}_{T}-{H}_{S}\right)/\left(1-{H}_{S}\right)$ and *H*_*T*_ represents the overall genetic diversity. In calculating *H*_*T*_ and *H*_*S*_, a correction was applied to account for the number of individuals for each species and each of the 999 × 4479 SNP data sets, using the *basic.stats* function (“hierfstat” R package^73^) in R v.4.2.1.

To investigate whether the 13 morphological traits used to compute intraspecific morphological disparity between geographic location were related to the spatial components of genetic diversity (i.e., *J*_*ST*_ and *J*_*S*_) obtained by applying a Hill number framework on genome-wide SNP data, we implemented a permutational multivariate analysis of variance (PERMANOVA) with 999 permutations with the *adonis2* function (R package “vegan”^74^). For the significant associations, each morphological trait disparity (i.e., ratio) was related individually as the response variable by means of a complete phylogenetic generalized least-squares (PGLS) regression using the *pgls* function (R package “caper”^75^).

### Congruence between intra- and interspecific morphological disparity

To evaluate the congruence between intra- and interspecific morphological disparity, we applied two complementary methods: a co-inertia analysis (CIA) and a phylogenetic regression method to account for phylogenetic dependency. First, we applied CIA to assess the congruence between the morphological trait variation at the species level (intraspecific disparity) and the family level (interspecific disparity). Since the datasets differed in dimensions, we replicated the interspecific data (family-level trait variation) for the number of species within each family, aligning it with the intraspecific dataset. In the CIA, the two data sets produced representations of the morphological variations in two hyperspaces. The CIA maximized the squared covariance between the projections of all the pairs of morphological trait variabilities on the co-inertia axes^76^. The congruence between the two data sets was measured using the *RV* coefficient of correlation which ranges from 0 (no correlation) to 1 (perfect correlation), with higher values indicating a stronger congruence between the data sets. Second, for each trait, we tested whether the individual relationships between the intraspecific and interspecific morphological disparity were robust to phylogenetic relatedness by using PGLS methods^77^. For this PGLS analysis, we pruned the phylogeny to include only the 17 species in our data set using the most comprehensive time-calibrated phylogeny of fishes^78^.

### Reporting summary

Further information on research design is available in the Nature Portfolio Reporting Summary linked to this article.

## Supplementary information

Supplementary material

Reporting summary

## Data Availability

The data files used to generate the genetic data are available from the Dryad Digital Repository: 10.5061/dryad.5qfttdz4d. Data related to fish morphology are available at: 10.16904/envidat.565

## Code availability

The scripts used to generate the genetic data are available from the Dryad Digital Repository: 10.5061/dryad.5qfttdz4d. The files and codes required to reproduce the results of this paper, are available at: https://github.com/Camillealbouy/Donati_et_al_COMMSBIO-23-3727 and 10.16904/envidat.565.
