# Supplementary material for: Continuity in morphological disparity in tropical reef fishes across evolutionary scales
Source: Commun Biol. 2025 Feb 17;8:252. doi: 10.1038/s42003-025-07634-7 (PMC11836192; doi:10.1038/s42003-025-07634-7)
Supplement: Supplementary file 1 — Supplementary material [file 42003_2025_7634_MOESM1_ESM.pdf]

Supplementary Information

Continuity in morphological disparity in tropical reef fishes across evolutionary scales

Supplementary Figure 1. Geographic location of the sampling sites in the Western Indian Ocean, where the intraspecific morphological data and the genetic data were collected. (a) Map of the sampling locations in the Western Indian Ocean: Mafia Island (Tanzania; 1), Mayotte Island (France island in Comorian archipelago; 2), the islands of Mahe, Praslin and La Digue (Seychelles; 3), and three central atolls of the Maldives, namely Kaafu North, Alif Alif, and Vaavu (Republic of Maldives; 4). The circled values on each small map indicate the number of species sampled at each location, and the red points mark the positions of the sampling sites at each location for the individuals considered in the genetic data set. (b) Standardized number of individuals selected for the genetic analyses, separated by species and location.

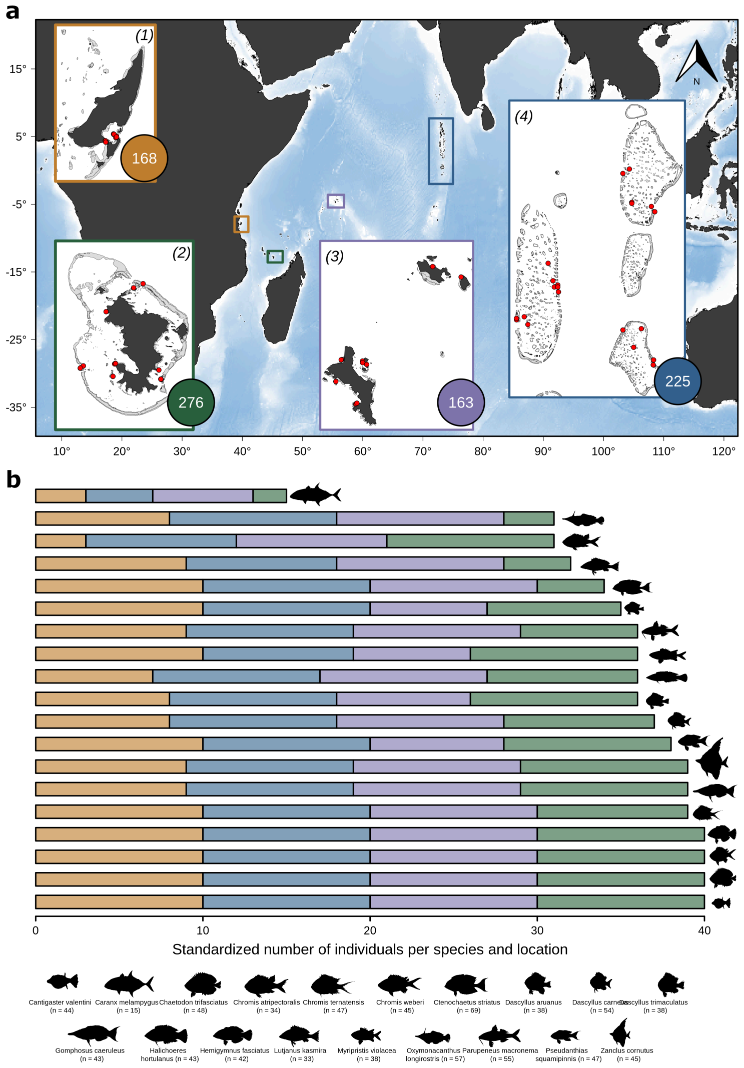

Supplementary Figure 2. Morphometric data collection on tropical reef fishes. The interspecific data set was constructed based on 1061 individual species (10 families) gathered from[1] and characterized by 14 available landmarks on the fish body (white dots). The intraspecific data set included 1111 individuals belonging to 17 species (embedded in the same 10 families); it was constructed based on the same 14 body landmarks plus 3 additional ones (orange dots). The intraspecific data set included only species belonging to families included in the interspecific data set. Landmarks around the head area are related to feeding mechanics, while the remaining ones are related to locomotion mechanics. Here, a male *Pseudanthias squamipinnis* (sea goldie, Serranidae) sample is shown as an example. For species with strong sexual dimorphism (e.g., *Pseudanthias squamipinnis*), only male specimens were included in the morphological trait analysis. All the morphological measurement terms are defined in the Supplementary Table 7.

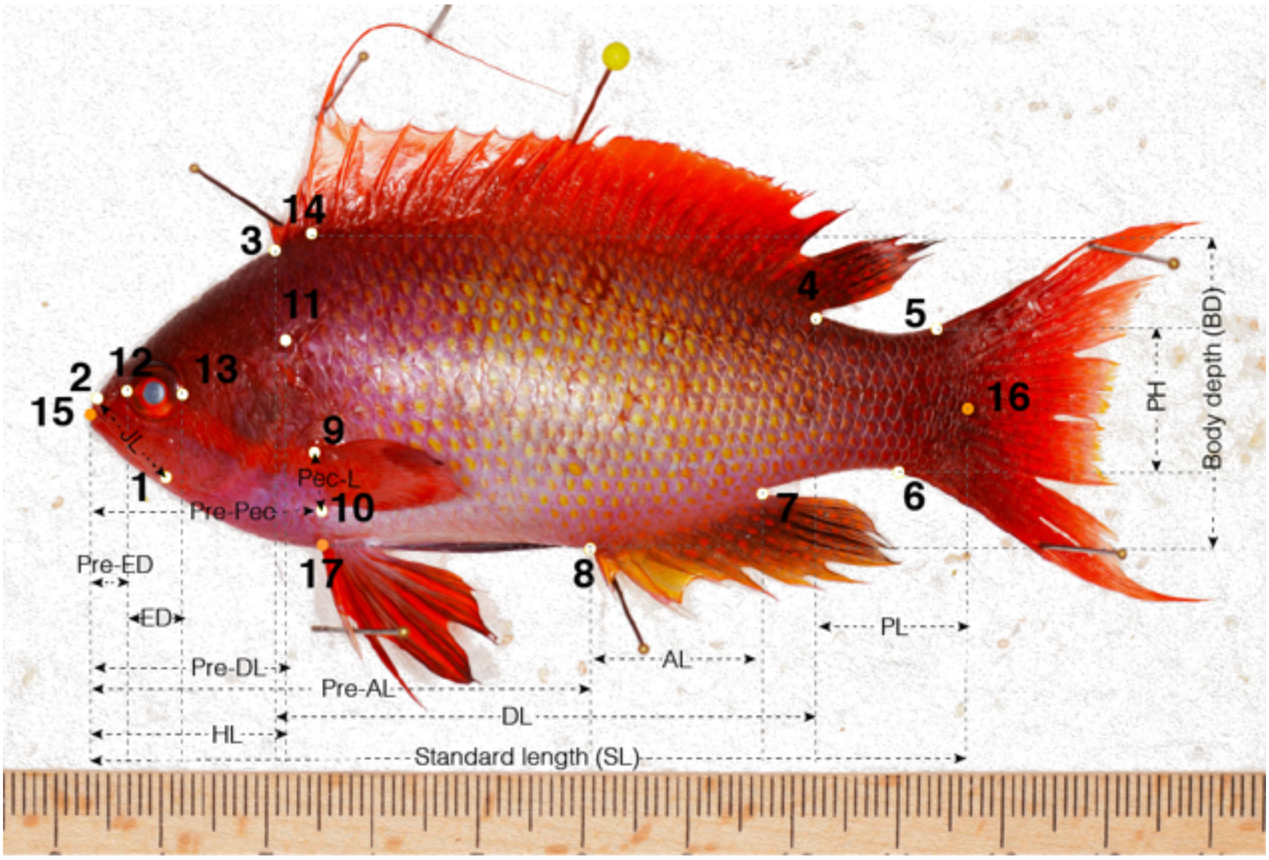

Reference:

1. Claverie, T. & Wainwright, P. C. A Morphospace for Reef Fishes: Elongation Is the Dominant Axis of Body Shape Evolution. PLoS One 9, e112732 (2014).

Supplementary Figure 3. Principal component analysis (PCA) based on body size and pelagic larval duration (PLD) for the 17 selected Western Indian Ocean fish species.

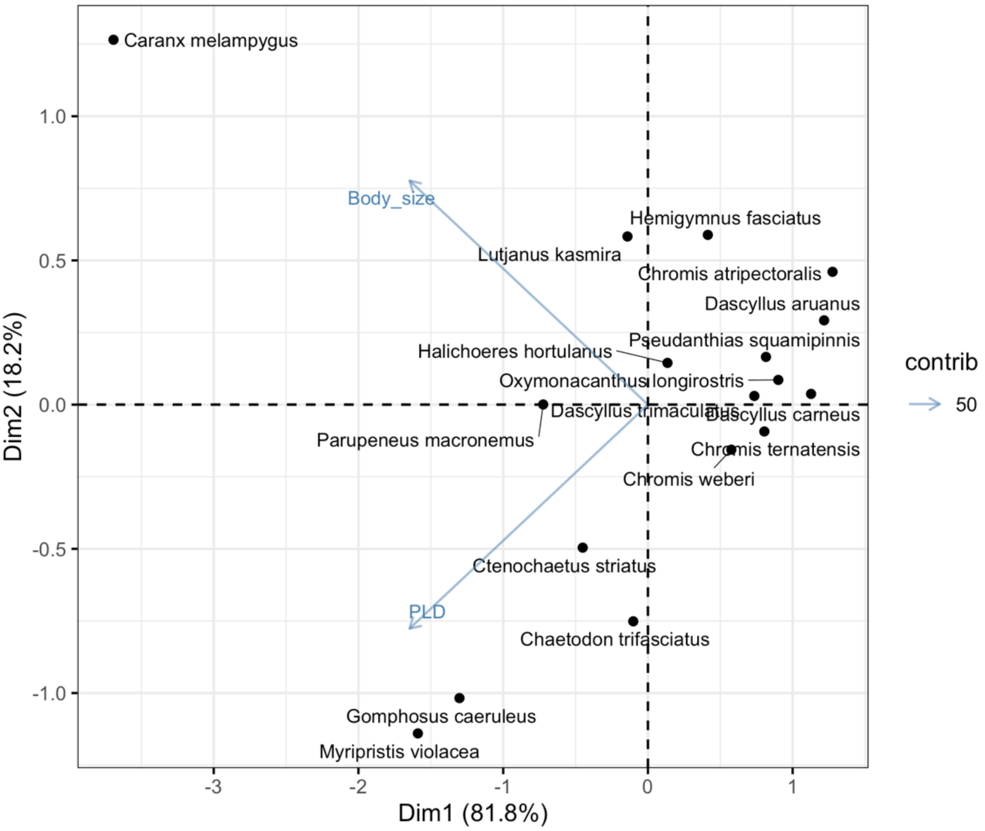

Supplementary Figure 4. Illustration of the morphological trait disparity (mtD) in tropical reef fishes at the intraspecific level. Each plot represents the morphological trait disparity for all the studied species: the black shaded areas represent the expected morphological variability under null models (999 randomizations), whereas the orange segments represent the values observed in our data set.

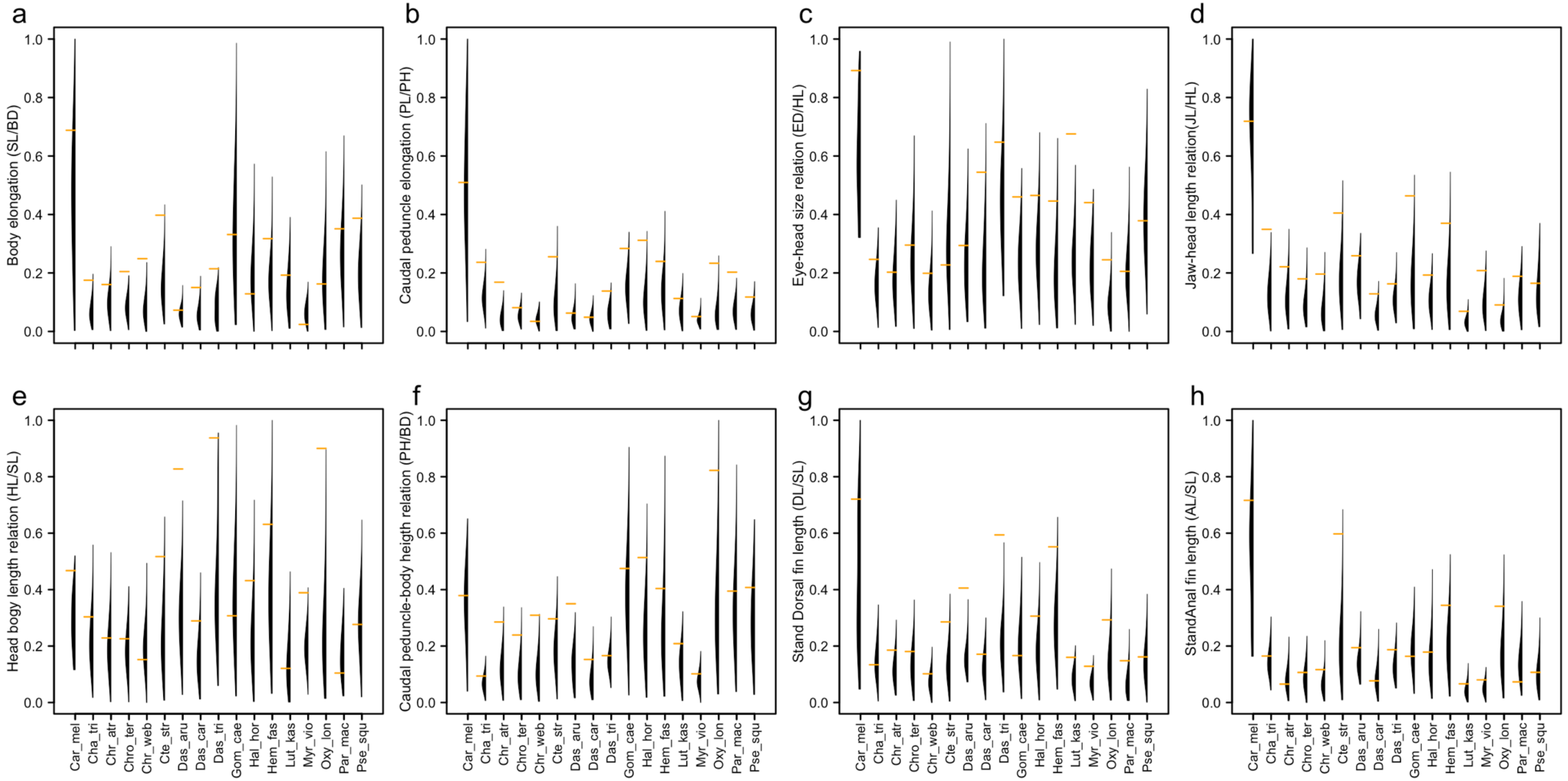

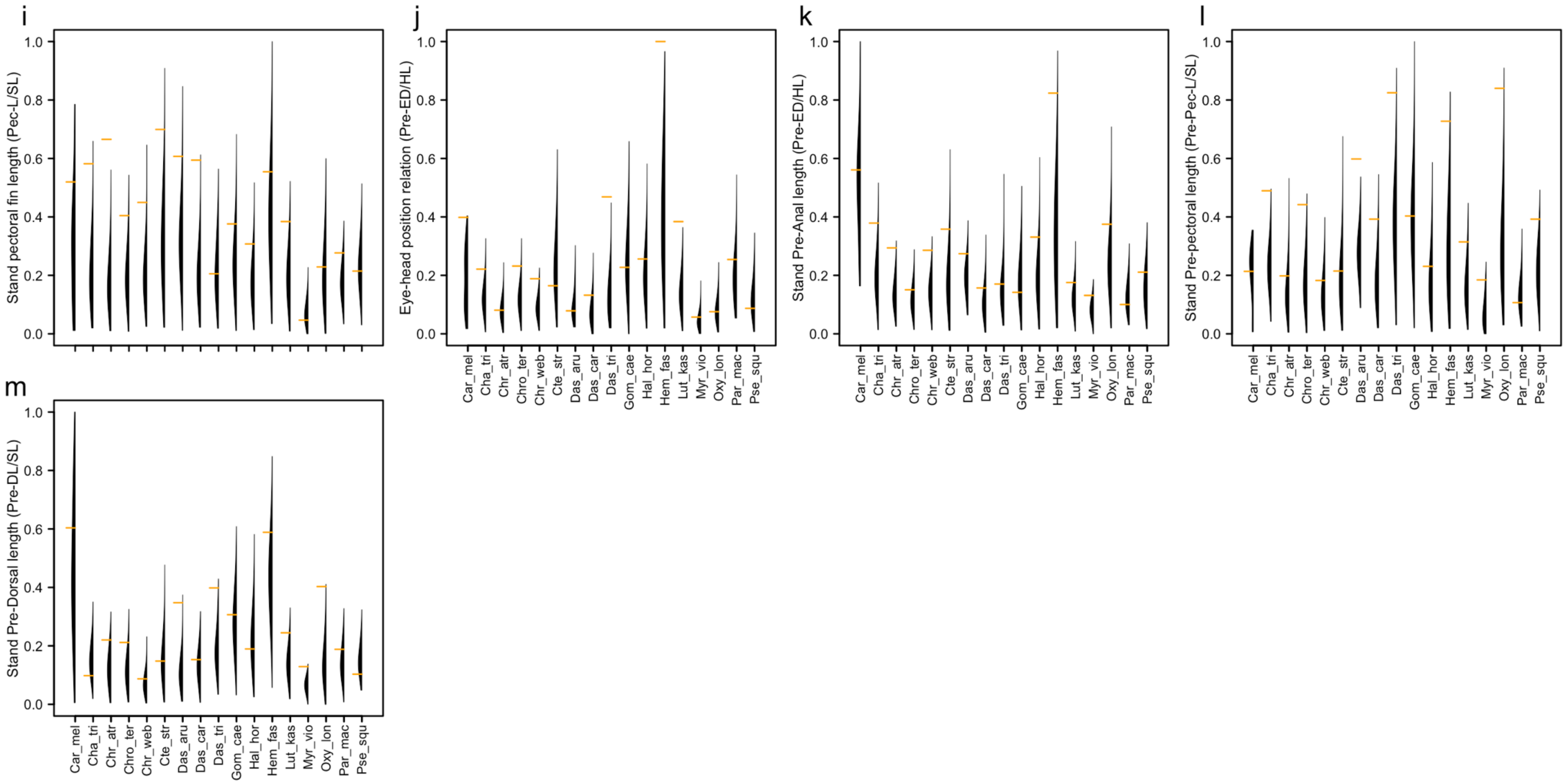
i
Stand pectoral fin length (Pec-L/SL)
j
Eye-head position relation (Pre-ED/HL)
k
Stand Pre-Anal length (Pre-ED/HL)
l
Stand Pre-pectoral length (Pre-Pec-L/SL)
m
Stand Pre-Dorsal length (Pre-DL/SL)
0.0
0.2
0.4
0.6
0.8
1.0
Car_mel
Cha_tri
Chr_atr
Chro_ter
Chr_web
Cte_str
Das_aru
Das_car
Das_tri
Gom_cae
Hal_hor
Hem_fas
Lut_kas
Myr_vio
Oxy_lon
Par_mac
Pse_squ

38

Supplementary Figure 5. Illustration of the morphological trait disparity (mtD) in tropical reef fishes at the interspecific level. Each plot represents the morphological trait disparity for all the studied families: the black shaded areas represent the expected morphological variability under null models (999 randomizations), whereas the blue segments represent the values observed in our data set.

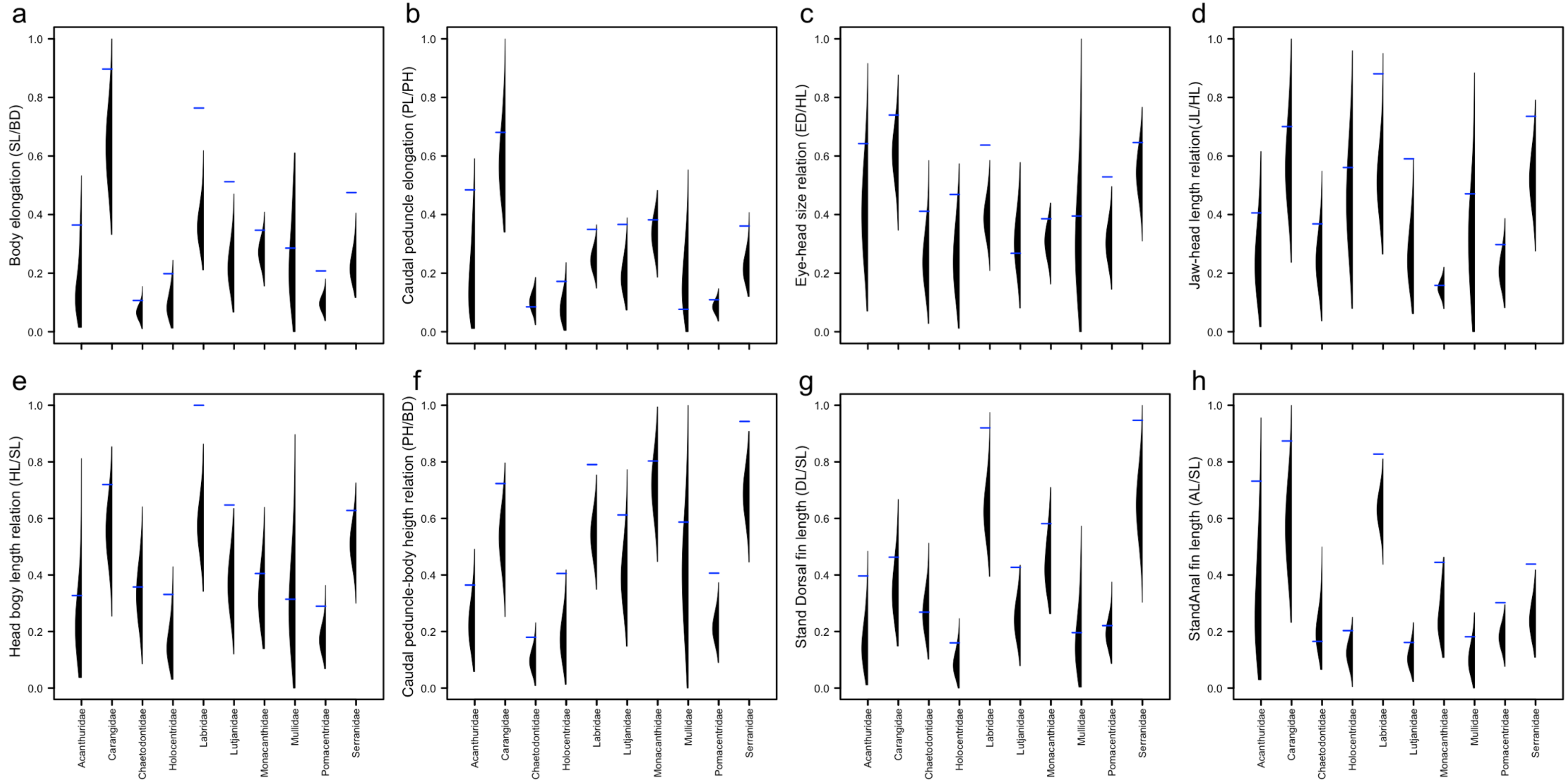

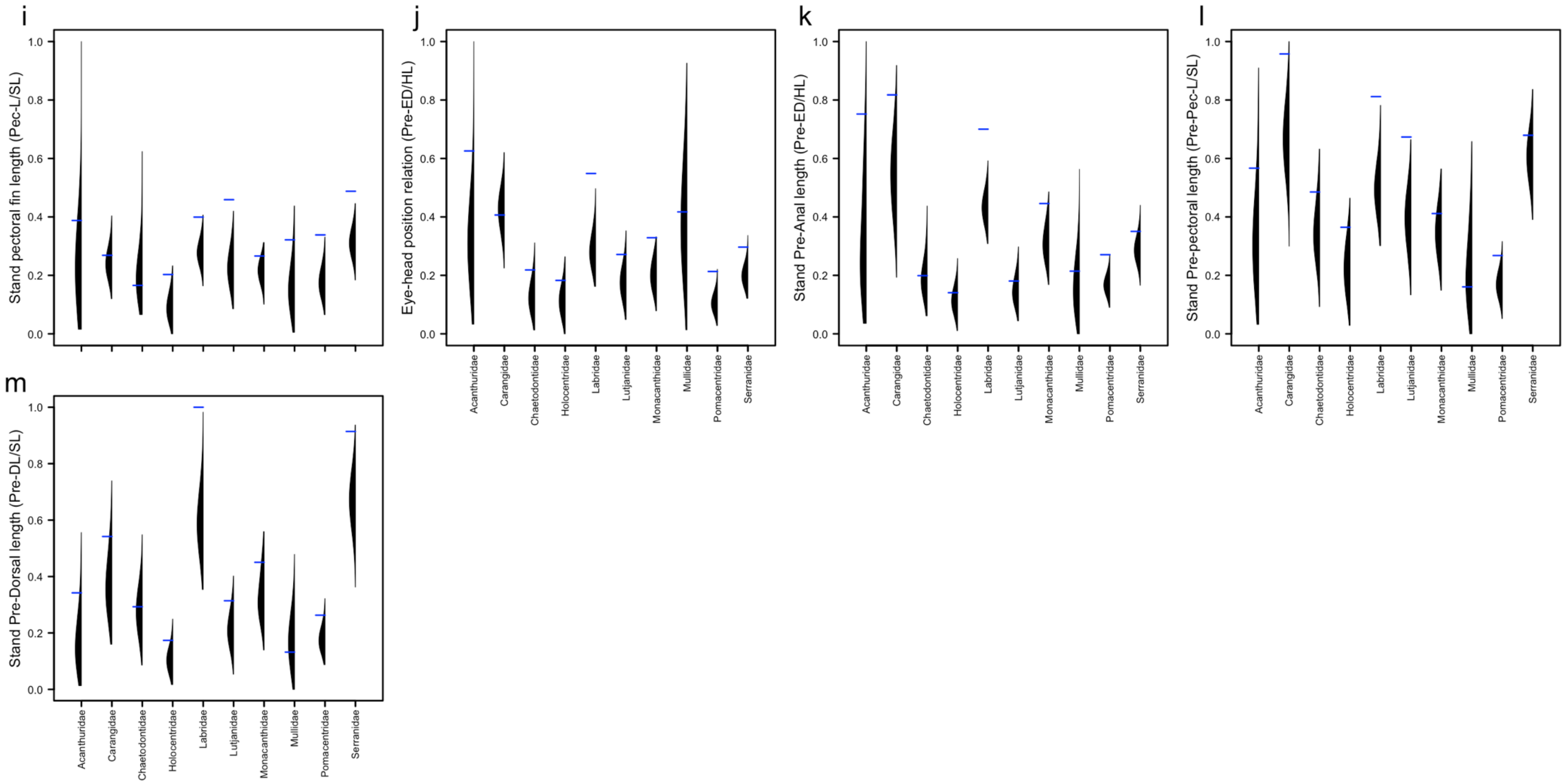
i
Stand pectoral fin length (Pec-L/SL)
j
Eye-head position relation (Pre-ED/HL)
k
Stand Pre-Anal length (Pre-ED/HL)
l
Stand Pre-pectoral length (Pre-Pec-L/SL)
m
Stand Pre-Dorsal length (Pre-DL/SL)
0.0
0.2
0.4
0.6
0.8
1.0
Acanthuridae
Carangidae
Chaetodontidae
Holocentridae
Labridae
Lutjanidae
Monacanthidae
Mullidae
Pomacentridae
Serranidae

Supplementary Figure 6. Intra- and interspecific morphological trait disparity (mtD) relationships for fish traits. Phylogenetic generalized least squares (PGLS) controlled relationships between the intra- and interspecific mtD in tropical reef fishes for (a) body elongation, relative (b) caudal peduncle elongation, (c) eye size, (d) head length, (e) dorsal fin size, (f) pectoral fin size, (g) eye position, (h) anal fin insertion, (i) pectoral fin insertion, and (j) dorsal fin insertion. The dashed line represents the Ordinary Least Square regression between intra- and interspecific morphological variability. The reported P-values in each panel corresponds to the significance of the relation accounting for phylogenetical relationship between species (PGLS: phylogenetic generalized least-squares). Each symbol contains two colors, one corresponding to the intraspecific data set and the other to the interspecific data set.

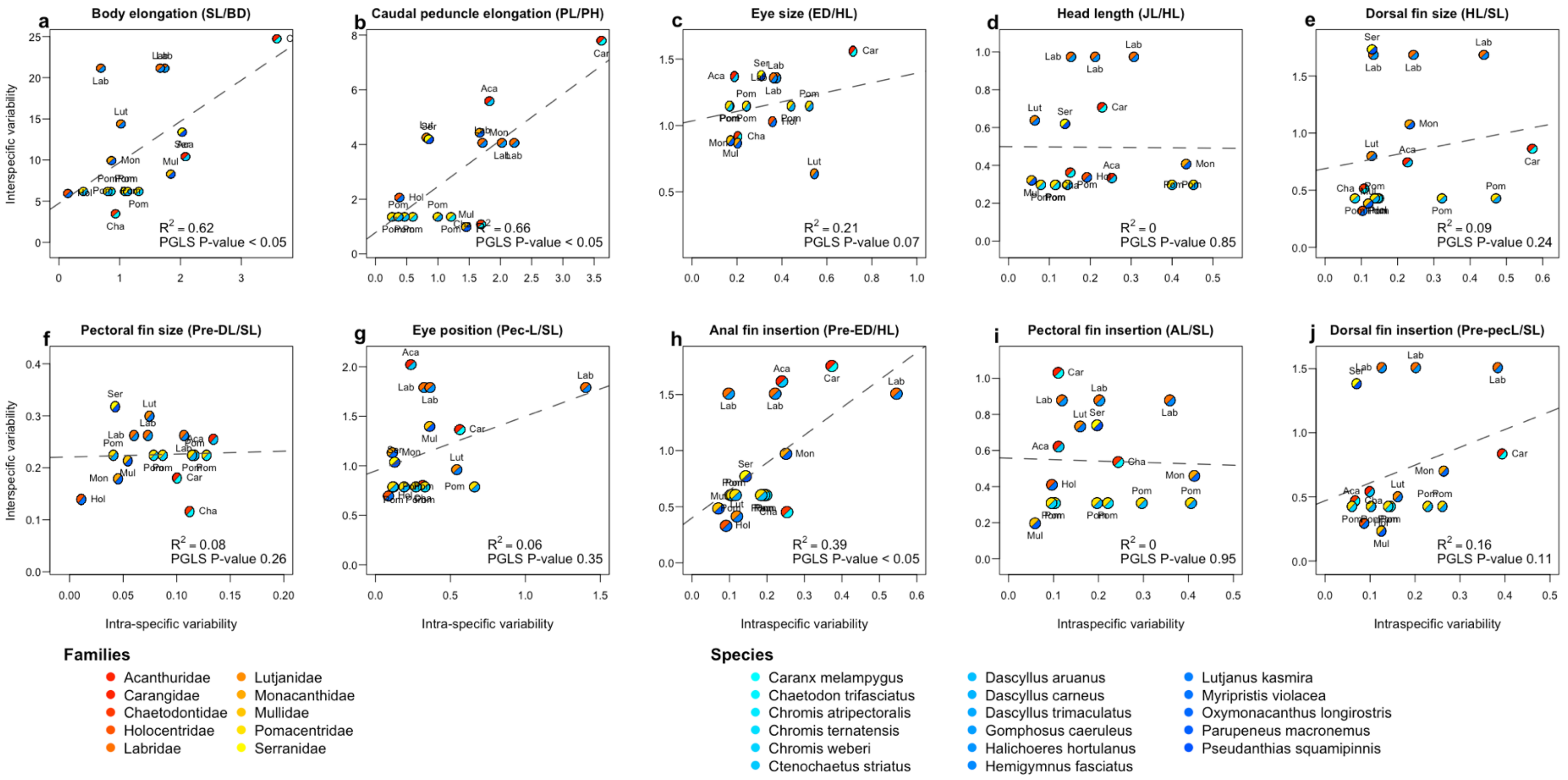

Supplementary Figure 7. Relationships between the intraspecific and interspecific morphological disparity in tropical reef fishes when excluding the Carangid fish species (*Caranx melampygus*). Relationships between intraspecific and interspecific variability for (a) caudal peduncle height (PL: caudal peduncle length, PH: caudal peduncle height); (b) anal fin length (AL: anal fin length) and (c) Jaw-head length relationship (JL: jaw length, HL: head length). The dashed line represents the Ordinary Least Square regression between intra- and interspecific morphological variability. The reported P-values in each panel corresponds to the significance of the relation accounting for phylogenetical relationship between species (PGLS: phylogenetic generalized least-squares). Each symbol contains two colors, one corresponding to the intraspecific data set and the other to the interspecific data set.

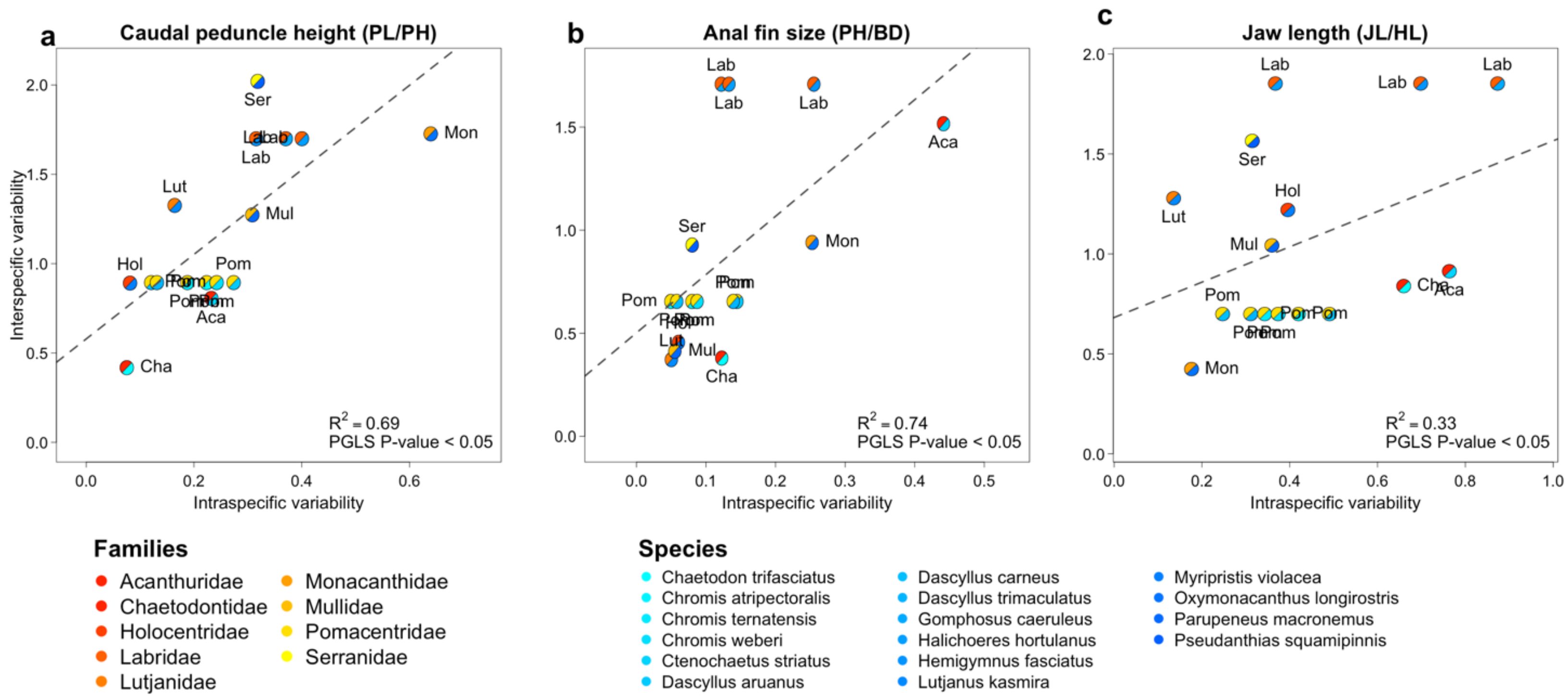

Supplementary Figure 8. Intra- and interspecific morphological trait disparity (mtD) relationships for fish traits when excluding the Carangid fish species (*Caranx melampygus*). Phylogenetic generalized least squares (PGLS) controlled relationships between the intra- and interspecific mtD in tropical reef fishes for for (a) body elongation, relative (b) caudal peduncle elongation, (c) eye size, (d) head length, (e) dorsal fin size, (f) pectoral fin size, (g) eye position, (h) anal fin insertion, (i) pectoral fin insertion, and (j) dorsal fin insertion. The dashed line represents the Ordinary Least Square regression between intra- and interspecific morphological variability. The reported P-values in each panel correspond to the significance of the relation accounting for the phylogenetic relationship between species (PGLS: phylogenetic generalized least-squares). Each symbol contains two colors, one corresponding to the intraspecific data set and the other to the interspecific data set.

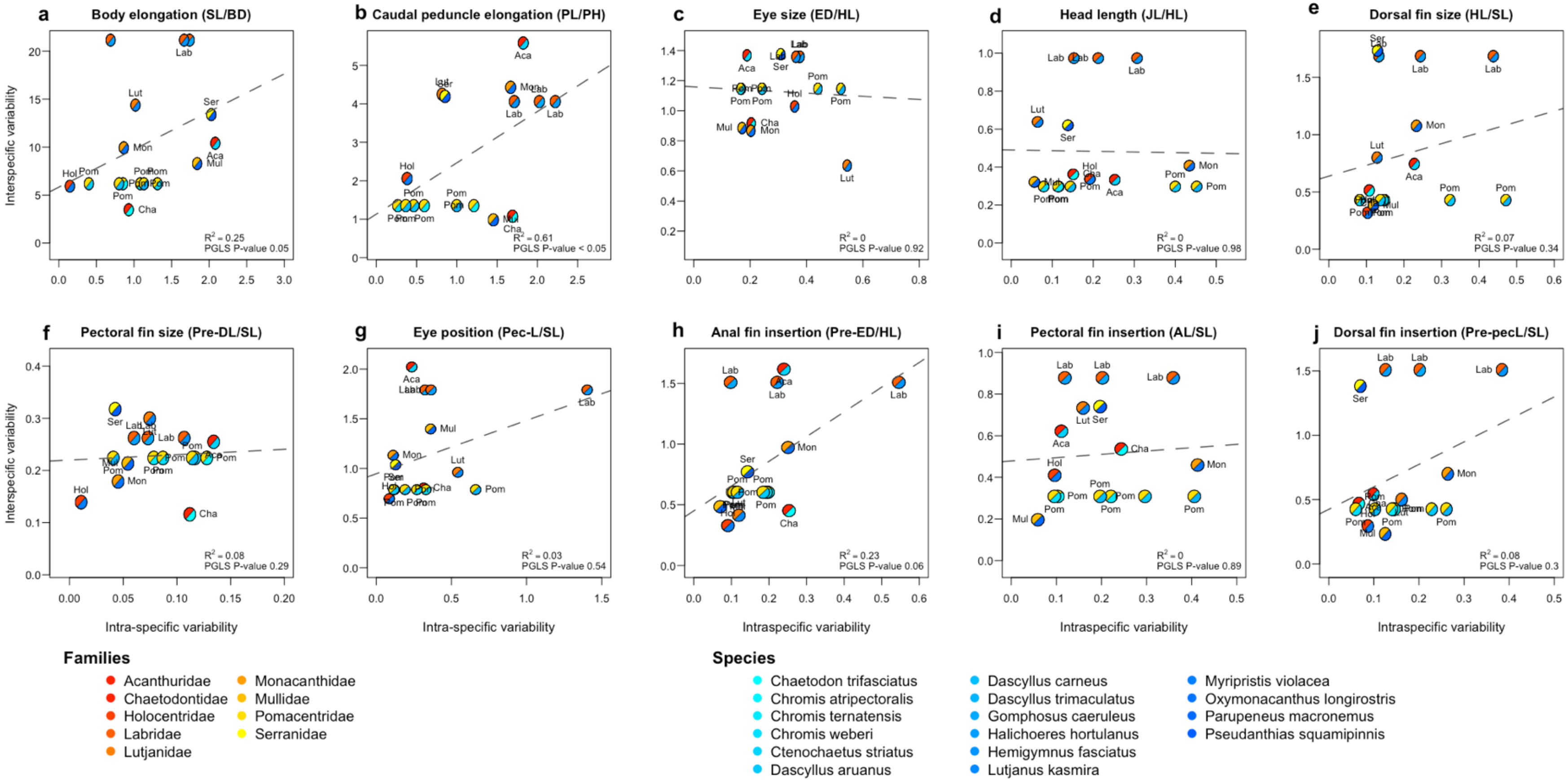

Supplementary Figure 9. Intraspecific morphological trait disparity (mtD) and genetic diversity (J_T) relationships for fish traits. Phylogenetic generalized least squares (PGLS) controlled relationships between the intraspecific mtD and the overall genetic diversity (J_T) for the relative (a) body elongation, (b) eye size, (c) jaw length, (d) head length, (e) caudal peduncle height, (f) dorsal fin size, (g) pectoral fin size, (h) eye position, (i) anal fin insertion, (h) eye position, (i) anal fin insertion and (j) pectoral fin insertion. The dashed line represents the Ordinary Least Square regression between total genetic diversity (J_T) and intraspecific morphological disparity. The reported P-values in each panel corresponds to the significance of the relation accounting for phylogenetical relationship between species (PGLS: phylogenetic generalized least-squares) Points are colored according to the interspecific data set, while labels indicate the intraspecific data set.

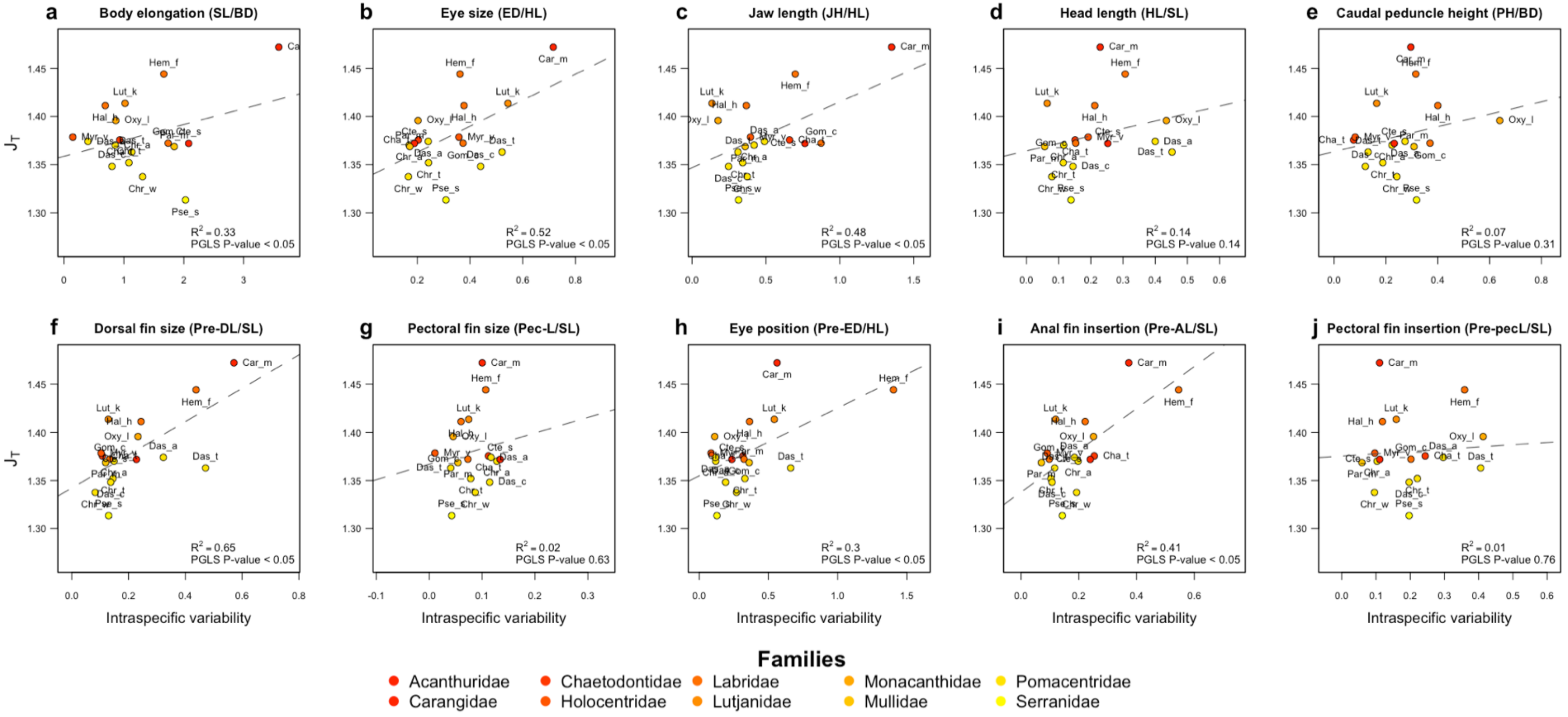

Supplementary Figure 10. Relationships between the total intraspecific genetic diversity (J_T) and the morphological trait variation for 16 tropical reef fish species when excluding the Carangid fish species, namely Caranx melampygus. Phylogenetic generalized least squares (PGLS) relationships between total intraspecific genetic diversity of the Western Indian Ocean and the variability in (a) relative anal fin size (AL: anal fin length; SL: standard length), (b) caudal peduncle elongation (PL: caudal peduncle length; PH: caudal peduncle height), and (c) the dorsal fin insertion (DL: dorsal fin length). The dashed line represents the Ordinary Least Square regression between total genetic diversity (J_T) and intraspecific morphological disparity. The reported P-values in each panel corresponds to the significance of the relation accounting for phylogenetical relationship between species (PGLS: phylogenetic generalized least-squares). Points are colored according to the interspecific data set, while labels indicate the intraspecific data set

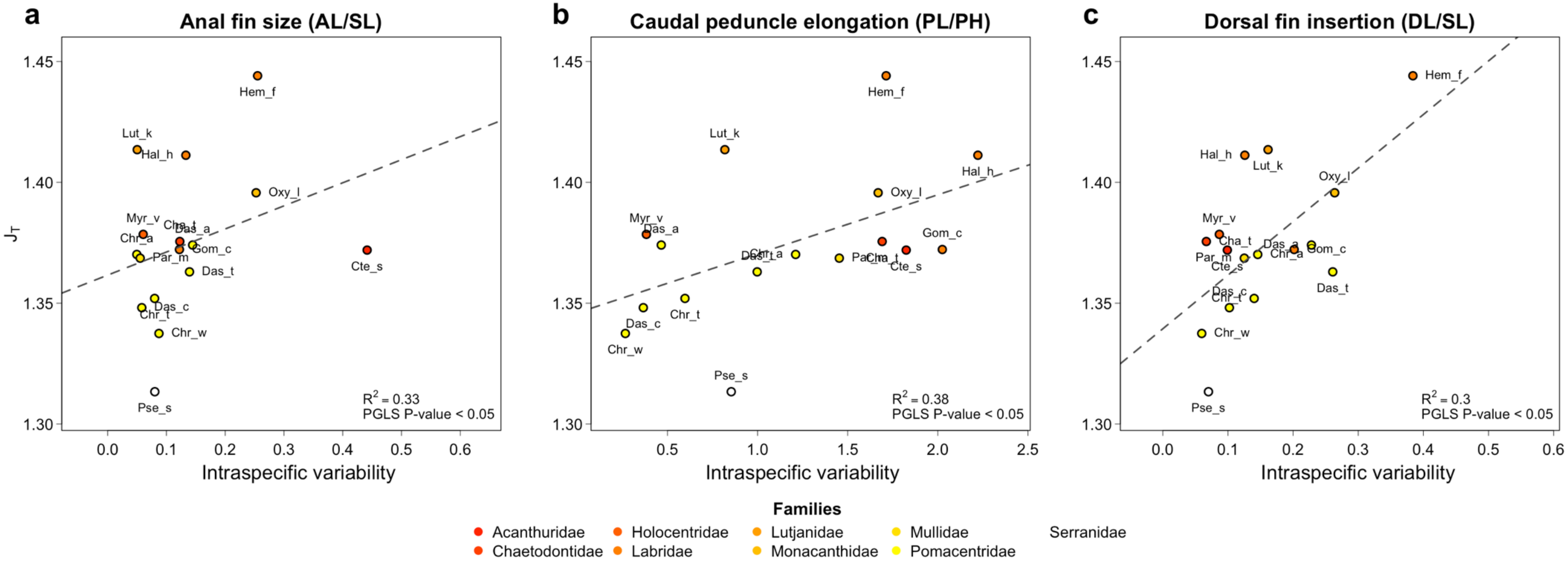

Supplementary Figure 11. Intraspecific morphological trait disparity (mtD) and genetic diversity (J_T) relationships for 16 tropical reef fish species when excluding the Carangid fish species, namely Caranx melampygus. Phylogenetic generalized least squares (PGLS) controlled relationships between the intraspecific mtD and the overall genetic diversity (J_T) for the relative (a) body elongation, (b) eye size, (c) jaw length, (d) head length, (e) caudal peduncle height, (f) dorsal fin size, (g) pectoral fin size, (h) eye position, (i) anal fin insertion, (j) pectoral fin insertion, and (j) dorsal fin insertion. The dashed line represents the Ordinary Least Square regression between total genetic diversity (J_T) and intraspecific morphological disparity. Points are colored according to the interspecific data set, while labels indicate the intraspecific data set.

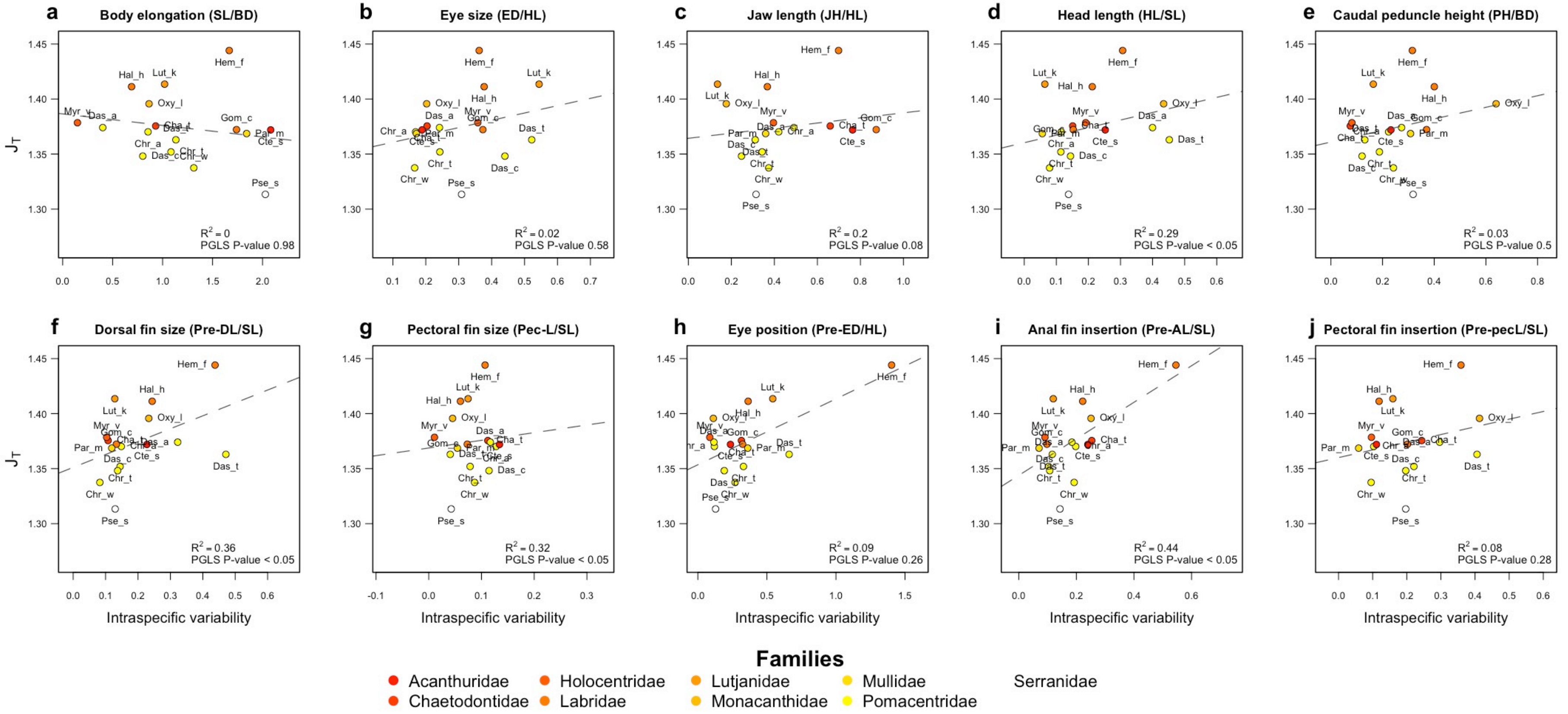

.

Supplementary Figure 12. Structural equation model (SEM) linking genetic diversity and morphological trait (mtD) disparity. SEM with dispersal trait BS (body size) and PLD (pelagic larval duration) included in a principal component analysis (PCA) axis and linked to γ diversity (Gamma) and β diversity (Beta). The relationships between the diversity components and intraspecific (PCoA_intra) and interspecific (PCoA_inter) trait disparity are also shown.

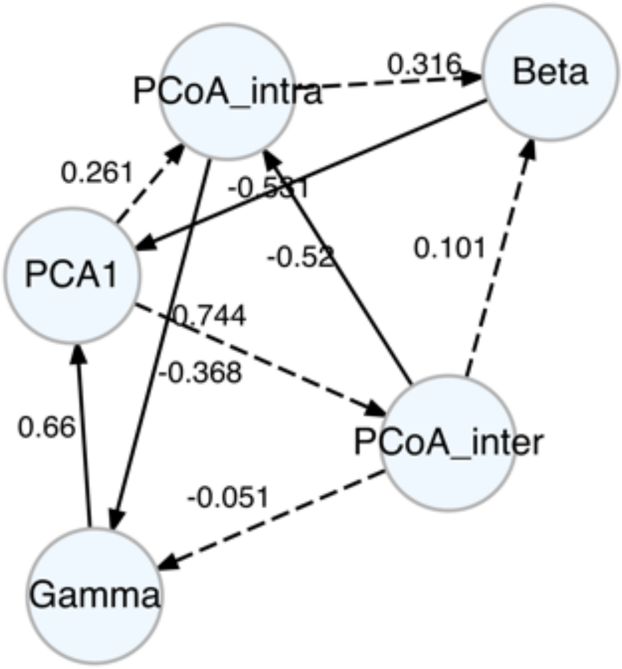

Supplementary Figure 13. Intra- and interspecific morphological trait disparity (mtD) relationships for fish traits by averaging the traits variability by fish family. Phylogenetic generalized least squares (PGLS) controlled relationships between the intra- and interspecific mtD in tropical reef fishes for (a) body elongation, relative (b) caudal peduncle elongation, (c) eye size, (d) jaw length, (e) head length, (f) caudal peduncle height, (g) dorsal fin size, (h) anal fin size, (i) pectoral fin size, (j) eye position, (k) anal fin insertion, (l) pectoral fin insertion, and (m) dorsal fin insertion. The dashed line represents the Ordinary Least Square regression between intra- and interspecific morphological variability. The reported P-values in each panel corresponds to the significance of the relation accounting for phylogenetical relationship between species (PGLS: phylogenetic generalized least-squares). Each symbol contains two colors, one corresponding to the intraspecific data set and the other to the interspecific data set.

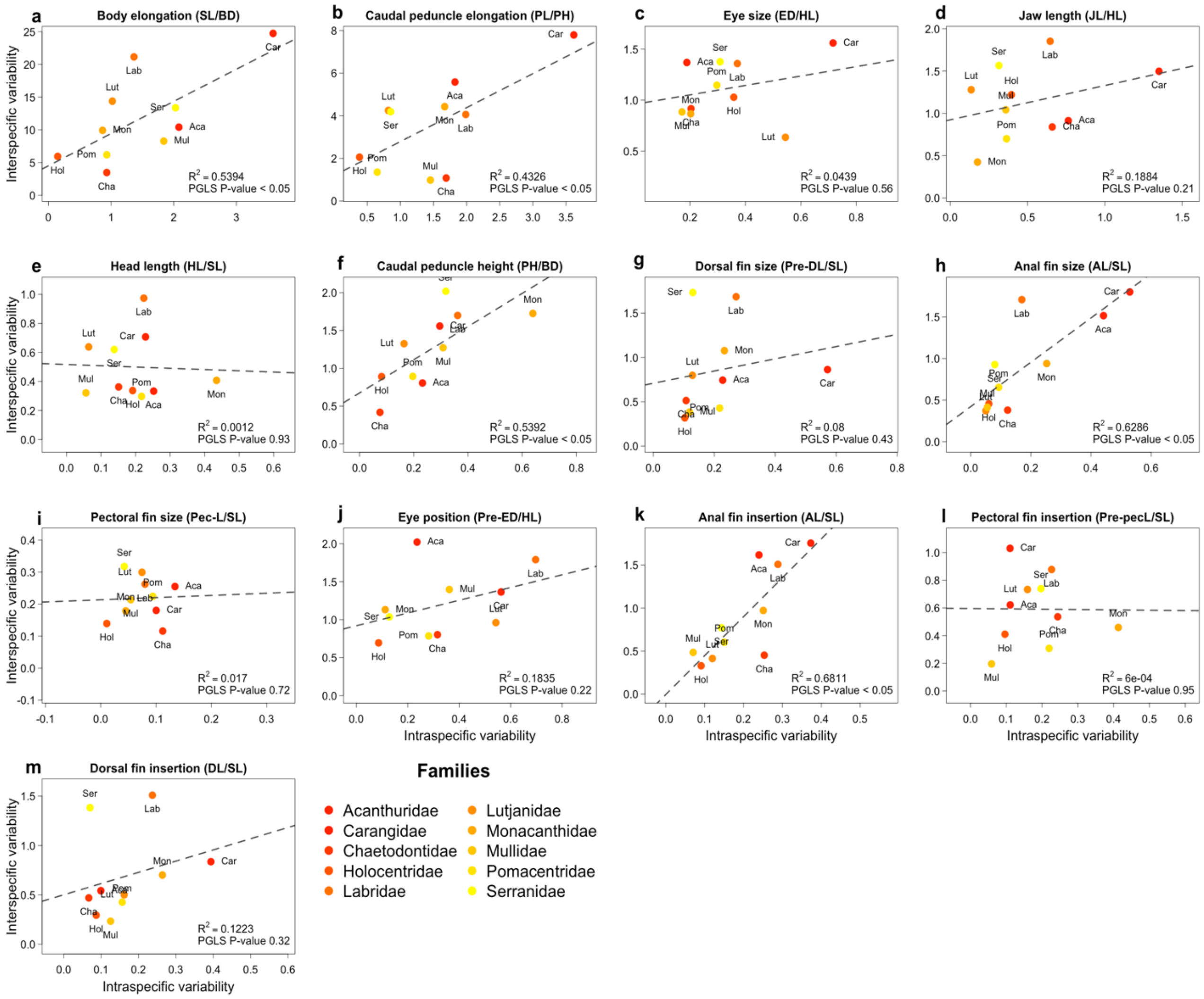

Supplementary Table 1. Ecological trait information for the 17 tropical reef fish species of the intraspecific data set. PLD: pelagic larval duration (days). Home range was coded as narrow for species with a restricted home range and wide for highly mobile species[1]. Diet was retrieved from Fishbase[2] and recoded according to the predominant diet composition, where PK stands for *plankton*, IS for *sessile invertebrates,* IM for *mobile benthic invertebrates,* OM for *omnivores*, and FC for *piscivores* (see Methods for details).

Family	Genus species	PLD (days)	Home range	Diet
Carangidae	*Caranx melampygus*	57.6	wide	FC
Chaetodontidae	*Chaetodon trifasciatus*	43	narrow	IS
Pomacentridae	*Chromis atripectoralis*	19	narrow	PK
Pomacentridae	*Chromis ternatensis*	28.5	narrow	PK
Pomacentridae	*Chromis weberi*	31.2	narrow	PK
Acanthuridae	*Ctenochaetus striatus*	43.87	narrow	OM
Pomacentridae	*Dascyllus aruanus*	21.1	narrow	PK
Pomacentridae	*Dascyllus carneus*	24.3	narrow	PK
Pomacentridae	*Dascyllus trimaculatus*	28	narrow	PK
Labridae	*Gomphosus caeruleus*	56.6	wide	IM
Labridae	*Halichoeres hortulanus*	32.5	wide	IM
Labridae	*Hemigymnus fasciatus*	25.8	wide	IM
Lutjanidae	*Lutjanus kasmira*	31	wide	IM
Holocentridae	*Myripristis violacea*	60.4	wide	PK
Monacanthidae	*Oxymonacanthus longirostris*	25.95	narrow	IS
Mullidae	*Parupeneus macronemus*	41.8	wide	IM
Serranidae	*Pseudanthias squamipinnis*	26	narrow	PK

Supplementary Table 2. The intraspecific data set (n = 1111 individuals, 17 species, 10 families). Specimens constituting this data set were collected in four regions of the Western Indian Ocean during 2016–2017. The table includes taxonomic information and the number of individuals sampled per population (i.e., Maldives, Mayotte Island, Seychelles and Mafia Island).

		Number of individuals (n)				
**Family**	**Genus species**	**Maldives**	**Mayotte**	**Seychelles**	**Mafia**	**Total**
Carangidae	*Caranx melampygus*	3	0	2	3	8
Chaetodontidae	*Chaetodon trifasciatus*	18	32	10	11	71
Pomacentridae	*Chromis atripectoralis*	15	53	14	9	91
Pomacentridae	*Chromis ternatensis*	15	47	11	10	83
Pomacentridae	*Chromis weberi*	18	71	15	11	115
Acanthuridae	*Ctenochaetus striatus*	26	40	6	12	84
Pomacentridae	*Dascyllus aruanus*	15	7	9	11	42
Pomacentridae	*Dascyllus carneus*	30	82	9	8	129
Pomacentridae	*Dascyllus trimaculatus*	11	7	9	11	38
Labridae	*Gomphosus caeruleus*	24	24	10	10	68
Labridae	*Halichoeres hortulanus*	28	33	10	10	81
Labridae	*Hemigymnus fasciatus*	11	12	11	6	40
Lutjanidae	*Lutjanus kasmira*	9	11	4	10	34
Holocentridae	*Myripristis violacea*	10	9	13	10	42
Monacanthidae	*Oxymonacanthus longirostris*	12	44	3	8	67
Mullidae	*Parupeneus macronemus*	23	23	9	11	66
Serranidae	*Pseudanthias squamipinnis*	19	8	11	14	52
						1111

Supplementary Table 3. Fish body landmarks and derived morphological traits. This table describes each biometric (a) measure and (b) ratio used in our analyses. To replace missing points in the interspecific (i.e., above species level) data set, the two most extreme points on the x axis were used as a measure of standard length (i.e., Landmark A; LMA: 15, Landmark B; LMB: 16), while the two most extreme points on the y axis were used as a measure of body depth (i.e., LM 14, 17). The linear distance measures (a; L1; L2) were converted into ratios (b; L1/L2) to serve as components of body, head and fin shapes. As with the procrustes-aligned body landmarks, the use of ratios reduces the chance that the analysis is dominated by a single variable, e.g., body size.

Type	Body part	LMA	LMB	Code	Biometric description
(a) Length	Head	2	1	JL - measure	Jaw length
		15	12	Pre-ED - measure	Pre ocular distance
		12	13	ED - measure	Eye diameter
		15	11	HL - measure	Head length
	Body	14	17	BD - measure	Body depth
		15	16	SL - measure	Standard length
		4	16	PL - measure	Caudal peduncle length
		4	7	PH - measure	Caudal peduncle height
	Fin	15	8	Pre-AL - measure	Anal fin anterior insertion
		8	7	AL - measure	Anal fin length
		15	3	Pre-DL - measure	Dorsal fin anterior insertion
		3	4	DL - measure	Dorsal fin length
		15	9	Pre-Pec-L - measure	Pectoral fin anterior insertion
		9	10	Pec-L - measure	Base of the pectoral fin length
**Type**	**Body part**	**L1**	**L2**	**Code**	**Biometric description**
(b) Ratio	Head	JL	HL	JL	Jaw–head-length relationship
		Pre-ED	HL	Pre-ED	Eye–head-position relationship
		ED	HL	ED	Eye–head-size relationship
		HL	SL	HL	Head–body-length relationship
	Body	SL	BD	Bod-Elo	Body elongation
		PL	PH	Ped-Elo	Caudal peduncle elongation
		PH	BD	Ped-H	Caudal-peduncle–body-height relationship
	Fin	Pre-AL	SL	Pre-AL	Pre-anal length standardized by standard length
		AL	SL	AL	Anal fin length standardized by standard length
		Pre-DL	SL	Pre-DL	Pre-dorsal fin length standardized by standard length
		DL	SL	DL	Dorsal fin length standardized by standard length
		Pre-Pec-L	SL	Pre-Pec-L	Pre-pectoral fin length standardized by standard length
		Pec-L	SL	Pec-L	Pectoral fin length standardized by standard length

Supplementary Table 4. Population sample size and single nucleotide polymorphisms (SNP) data on 17 tropical reef fish species. The table includes information on the total sampling success per sampling sites (MF: Mafia Island; MV: Maldives; MY: Mayotte Island; SC: Seychelles), as well as the number of raw SNPs and filtered SNPs used in the downstream analyses (see genotyping in Methods). To compute genetic diversity metrics across the 17 species, the SNP data had to be down-sampled to the lowest common number of filtered SNPs and number of individuals per population standardized over 999 randomizations (see genotyping and computation of genetic diversity metrics in Methods).

		**Total sampling**						**Standardized resampling 999×**				
**Genus species**	**Species code**	**MF**	**MV**	**MY**	**SC**	**Total SNPs**	**Filtered SNPs**	**MF**	**MV**	**MY**	**SC**	**Resampled SNPs**
Caranx melampygus	Car_m	3	4	6	2	107395	15431	3	4	6	2	4479
Chaetodon trifasciatus	Cha_t	11	12	15	10	327595	20950	10	10	10	10	4479
Chromis atripectoralis	Chr_a	3	9	9	13	1255718	7225	3	9	9	10	4479
Chromis ternatensis	Chr_t	10	14	14	9	873075	17369	10	10	10	9	4479
Chromis weberi	Chr_w	10	10	13	12	926904	14370	10	10	10	10	4479
Ctenochaetus striatus	Cte_s	10	15	40	4	697607	9664	10	10	10	4	4479
Dascyllus aruanus	Das_a	11	12	7	8	986800	22105	10	10	7	8	4479
Dascyllus carneus	Das_c	8	17	20	9	1086702	16260	8	10	10	9	4479
Dascyllus trimaculatus	Das_t	8	12	8	10	682522	38934	8	10	8	10	4479
Gomphosus caeruleus	Gom_c	9	12	12	10	854088	6253	9	10	10	10	4479
Halichoeres hortulanus	Hal_h	7	14	13	9	242929	18934	7	10	10	9	4479
Hemigymnus fasciatus	Hem_f	10	11	11	10	229050	15606	10	10	10	10	4479
Lutjanus kasmira	Lut_k	9	9	11	4	806613	7420	9	9	10	4	4479
Myripristis violacea	Myr_v	10	9	7	12	463771	14890	10	9	7	10	4479
Oxymonacanthus longirostris	Oxy_l	8	11	35	3	111506	9679	8	10	10	3	4479
Parupeneus macronema	Par_m	9	17	22	7	635207	7432	9	10	10	7	4479
Pseudanthias squamipinnis	Pse_s	13	15	8	11	696705	4479	10	10	8	10	4479

Supplementary Table 5. The interspecific data set (n = 1061 species, 10 families). Specimens constituting this data set were extracted from [3]. The table includes taxonomic information and the number of species within each genus for the 10 tropical reef fish families that are also found in the intraspecific data set.

Genus	n	Family	Genus	n	Family	Genus	n	Family	Genus	n	Family	Genus	n	Family
Acanthurus	29		*Myripristis*	17		*Pseudocoris*	4		*Stephanolepis*	1	Monacanthidae	*Cromileptes*	1	
Ctenochaetus	8		*Neoniphon*	4		*Pseudodax*	1		*Thamnaconus*	3		*Dermatolepis*	1	
Naso	12	Acanthuridae	*Ostichthys*	5	Holocentridae	*Pseudojuloides*	7		*Mulloidichthys*	2		*Diploprion*	2	
Paracanthurus	1		*Plectrypops*	1		*Pseudolabrus*	8		*Parupeneus*	20	Mullidae	*Epinephelus*	55	
Prionurus	3		*Pristilepis*	1		*Pteragogus*	7		*Upeneichthys*	1		*Gracila*	1	
Zebrasoma	6		*Sargocentron*	24		*Stethojulis*	7	Labridae	*Upeneus*	13		*Grammistes*	1	
Alectis	2		*Ammolabrus*	1		*Suezichthys*	2		*Abudefduf*	13		*Grammistops*	1	
Alepes	5		*Anampses*	12		*Thalassoma*	17		*Acanthochromis*	1		*Holanthias*	8	
Atule	1		*Bodianus*	15		*Wetmorella*	2		*Amblyglyphidodon*	4		*Hypoplectrodes*	2	
Carangoides	12		*Cheilinus*	7		*Xiphocheilus*	1		*Amblypomacentrus*	2		*Liopropoma*	10	
Caranx	6		*Cheilio*	1		*Xyrichtys*	11		*Amphiprion*	21		*Luzonichthys*	3	
Decapterus	3		*Choerodon*	15		*Aphareus*	2		*Cheiloprion*	1		*Nemanthias*	1	Serranidae
Elagatis	1		*Cirrhilabrus*	25		*Aprion*	1		*Chromis*	53		*Plectranthias*	11	
Gnathanodon	1		*Conniella*	1		*Etelis*	2		*Chrysiptera*	23		*Plectropomus*	7	
Megalaspis	1		*Coris*	16		*Lipocheilus*	1		*Dascyllus*	9		*Pogonoperca*	1	
Parastromateus	1	Carangidae	*Cymolutes*	3		*Lutjanus*	35		*Dischistodus*	5		*Pseudanthias*	43	
Pseudocaranx	2		*Diproctacanthus*	1		*Macolor*	1	Lutjanidae	*Hemiglyphidodon*	1	Pomacentridae	*Pseudogramma*	6	
Scomberoides	3		*Epibulus*	2		*Paracaesio*	4		*Lepidozygus*	1		*Rabaulichthys*	1	
Selar	1		*Gomphosus*	2		*Pinjalo*	2		*Neoglyphidodon*	8		*Rainfordia*	1	
Selaroides	1		*Halichoeres*	44		*Pristipomoides*	8		*Neopomacentrus*	12		*Sacura*	2	
Seriola	3		*Hemigymnus*	2	Labridae	*Symphorichthys*	1		*Parma*	2		*Serranocirrhitus*	1	
Seriolina	1		*Hologymnosus*	3		*Symphorus*	1		*Plectroglyphidodon*	9		*Suttonia*	1	
Trachinotus	6		*Labrichthys*	1		*Acreichthys*	1		*Pomacentrus*	34		*Trachypoma*	1	
Trachurus	1		*Labroides*	5		*Aluterus*	1		*Pomachromis*	3		*Variola*	2	
Ulua	1		*Labropsis*	6		*Amanses*	1		*Premnas*	1				
Amphichaetodon	2		*Larabicus*	1		*Brachaluteres*	1		*Pristotis*	2				
Chaetodon	73		*Leptojulis*	4		*Cantherhines*	7		*Stegastes*	11				
Chelmon	3		*Macropharyngodon*	8		*Meuschenia*	1		*Acanthistius*	1				
Chelmonops	1		*Novaculichthys*	4		*Monacanthus*	2	Monacanthidae	*Aethaloperca*	1				
Coradion	3		*Oxycheilinus*	7		*Oxymonacanthus*	2		*Anyperodon*	1				
Forcipiger	2	Chaetodontidae	*Paracheilinus*	8		*Paraluteres*	1		*Aporops*	1				
Hemitaurichthys	3		*Parajulis*	1		*Paramonacanthus*	3		*Aulacocephalus*	1	Serranidae			
Heniochus	7		*Polylepion*	1		*Pervagor*	8		*Belonoperca*	2				
Parachaetodon	1		*Pseudocheilinops*	1		*Pseudalutarius*	1		*Caprodon*	2				
Prognathodes	1		*Pseudocheilinus*	4		*Pseudomonacanthus*	1		*Cephalopholis*	18			**Total species**	**1061**

Supplementary Table 6. Values of multiplicative and additive partitioning frameworks of genetic diversity. To quantify the different $\overline{\alpha}$, β and γ components we used the partitioning for true diversities proposed by [4] and applied to genetic data, expressed as follows: $J_T = J_S \times J_{ST}$, where J_S represents the within-population genetic component ($\overline{\alpha}$), J_{ST} represents the between-population (β) component, and J_T represents the overall genetic diversity (γ). All of these metrics were calculated for each species and each of the 999 × 4479 SNP data sets.

**Genus species**	J_T **(γ)**	J_S **($\overline{\alpha}$)**	J_{ST} **(β)**
Canthigaster valentini	1.3785	1.3743	1.0030
Caranx melampygus	1.4721	1.4739	1.0000
Chaetodon trifasciatus	1.3755	1.3735	1.0015
Chromis atripectoralis	1.3702	1.3655	1.0034
Chromis ternatensis	1.3520	1.3459	1.0045
Chromis weberi	1.3375	1.3317	1.0043
Ctenochaetus striatus	1.3720	1.3717	1.0002
Dascyllus aruanus	1.3741	1.3654	1.0064
Dascyllus carneus	1.3482	1.3466	1.0011
Dascyllus trimaculatus	1.3630	1.3615	1.0011
Gomphosus caeruleus	1.3722	1.3716	1.0004
Halichoeres hortulanus	1.4112	1.4097	1.0011
Hemigymnus fasciatus	1.4441	1.4241	1.0140
Lutjanus kasmira	1.4135	1.4131	1.0003
Myripristis violacea	1.3785	1.3783	1.0001
Oxymonacanthus longirostris	1.3957	1.3782	1.0127
Parupeneus macronemus	1.3686	1.3625	1.0045
Pseudanthias squamipinnis	1.3134	1.3099	1.0026
