# Supplementary material for: Continuity in morphological disparity in tropical reef fishes across evolutionary scales
Source: Commun Biol. 2025 Feb 17;8:252. doi: 10.1038/s42003-025-07634-7 (PMC11836192; doi:10.1038/s42003-025-07634-7)
Supplement: Supplementary file 2 — Reporting summary [file 42003_2025_7634_MOESM2_ESM.pdf]

Corresponding author(s): camille albouy

Last updated by author(s): 2024/07/05

Reporting Summary

Nature Portfolio wishes to improve the reproducibility of the work that we publish. This form provides structure for consistency and transparency in reporting. For further information on Nature Portfolio policies, see our Editorial Policies and the Editorial Policy Checklist.

Statistics

For all statistical analyses, confirm that the following items are present in the figure legend, table legend, main text, or Methods section.

n/a	Confirmed	
☐	☒	The exact sample size (*n*) for each experimental group/condition, given as a discrete number and unit of measurement
☐	☒	A statement on whether measurements were taken from distinct samples or whether the same sample was measured repeatedly
☐	☒	The statistical test(s) used AND whether they are one- or two-sided<br>*Only common tests should be described solely by name; describe more complex techniques in the Methods section.*
☐	☒	A description of all covariates tested
☐	☒	A description of any assumptions or corrections, such as tests of normality and adjustment for multiple comparisons
☐	☒	A full description of the statistical parameters including central tendency (e.g. means) or other basic estimates (e.g. regression coefficient) AND variation (e.g. standard deviation) or associated estimates of uncertainty (e.g. confidence intervals)
☐	☒	For null hypothesis testing, the test statistic (e.g. *F*, *t*, *r*) with confidence intervals, effect sizes, degrees of freedom and *P* value noted<br>*Give P values as exact values whenever suitable.*
☒	☐	For Bayesian analysis, information on the choice of priors and Markov chain Monte Carlo settings
☒	☐	For hierarchical and complex designs, identification of the appropriate level for tests and full reporting of outcomes
☐	☒	Estimates of effect sizes (e.g. Cohen's *d*, Pearson's *r*), indicating how they were calculated

Our web collection on statistics for biologists contains articles on many of the points above.

Software and code

Policy information about availability of computer code

Data collection	No software was used to collect the data.
Data analysis	We mainly used R version 4.2.1 and employed the basic.stats function from the hierfstat R package, the adonis2 function from the vegan R package, and a function from the caper R package. To analyses the fish images, we used the tpsUtil32and the tpsDIG v.3 software.

For manuscripts utilizing custom algorithms or software that are central to the research but not yet described in published literature, software must be made available to editors and reviewers. We strongly encourage code deposition in a community repository (e.g. GitHub). See the Nature Portfolio guidelines for submitting code & software for further information.

Data

Policy information about availability of data

All manuscripts must include a data availability statement. This statement should provide the following information, where applicable:

- Accession codes, unique identifiers, or web links for publicly available datasets
- A description of any restrictions on data availability
- For clinical datasets or third party data, please ensure that the statement adheres to our policy

The data and the simulation results that support the findings of this study are available in Figshare with the identifier https://doi.orndbjsg/XXXX

Research involving human participants, their data, or biological material

Policy information about studies with human participants or human data. See also policy information about sex, gender (identity/presentation), and sexual orientation and race, ethnicity and racism.

Reporting on sex and gender	*Use the terms sex (biological attribute) and gender (shaped by social and cultural circumstances) carefully in order to avoid confusing both terms. Indicate if findings apply to only one sex or gender; describe whether sex and gender were considered in study design; whether sex and/or gender was determined based on self-reporting or assigned and methods used. Provide in the source data disaggregated sex and gender data, where this information has been collected, and if consent has been obtained for sharing of individual-level data; provide overall numbers in this Reporting Summary. Please state if this information has not been collected. Report sex- and gender-based analyses where performed, justify reasons for lack of sex- and gender-based analysis.*
Reporting on race, ethnicity, or other socially relevant groupings	*Please specify the socially constructed or socially relevant categorization variable(s) used in your manuscript and explain why they were used. Please note that such variables should not be used as proxies for other socially constructed/relevant variables (for example, race or ethnicity should not be used as a proxy for socioeconomic status). Provide clear definitions of the relevant terms used, how they were provided (by the participants/respondents, the researchers, or third parties), and the method(s) used to classify people into the different categories (e.g. self-report, census or administrative data, social media data, etc.) Please provide details about how you controlled for confounding variables in your analyses.*
Population characteristics	*Describe the covariate-relevant population characteristics of the human research participants (e.g. age, genotypic information, past and current diagnosis and treatment categories). If you filled out the behavioural & social sciences study design questions and have nothing to add here, write "See above."*
Recruitment	*Describe how participants were recruited. Outline any potential self-selection bias or other biases that may be present and how these are likely to impact results.*
Ethics oversight	*Identify the organization(s) that approved the study protocol.*

Note that full information on the approval of the study protocol must also be provided in the manuscript.

Field-specific reporting

Please select the one below that is the best fit for your research. If you are not sure, read the appropriate sections before making your selection.

☐ Life sciences ☐ Behavioural & social sciences ☒ Ecological, evolutionary & environmental sciences

For a reference copy of the document with all sections, see nature.com/documents/nr-reporting-summary-flat.pdf

Ecological, evolutionary & environmental sciences study design

All studies must disclose on these points even when the disclosure is negative.

Study description	We combined measurements of genetic diversity with data on body morphology from multiple geographic locations to test whether dispersal ability plays a major role in shaping morphological disparity in tropical reef fishes at both intra- and interspecific levels. We selected 17 tropical reef fish species from 10 common families, with varying adult body sizes and pelagic larval durations. We sampled a total of 1,111 individuals of these species from four locations across the Western Indian Ocean. We measured 13 morphological traits per individual based on distances between landmarks at specific locations on the fish body. We used these ratios to compute morphological disparity between geographic locations (intraspecific level), which we compared between the genera of the corresponding families (interspecific level).
Research sample	We selected 17 tropical reef fishes species, from 10 common families, with varying adult body size and pelagic larval duration corresponding to a gradient of dispersal ability. We computed the interspecific disparity using a morphometric data set including 1061 Indo-Pacific reef fish species from the 10 same families from (Claverie & Wainwrigh 2014). This "interspecific" data set included one adult individual per species, with measurements made on photographs from Dr. Jack Randall (Bishop Museum, Honolulu, Hawaii).<br><br>1. Claverie, T. & Wainwright, P. C. Morphospace for Reef Fishes: Elongation Is the Dominant Axis of Body Shape Evolution. PLoS One 9, e112732 (2014).
Sampling strategy	Sampling was conducted using hand barrier nets while scuba diving, in compliance with local regulations. For the largest-bodied target species (e.g., Caranx melampygus), additional specimens were collected from local fish markets, where the fishing location of the fish was known. When possible, we sampled 10 individuals per species per location, which is enough samples to explore genetic differentiation between distant locations.
Data collection	All the co-authors participated in the collection and processing of data. Each specimen was pinned head facing left on a board to provide the most accurate view of the full body shape, including fin extensions. To overcome occasional post-mortem rigidity deformations, the epaxial muscle was massaged. Each specimen was photographed together with a scale bar using a digital single-

lens reflex camera. An AF Micro-NIKKOR 60 mm F/2.8D lens (Nikon) was used to avoid distortion.

After photographing, we sampled muscle tissues from each individual of the 17 target species collected from all four geographically isolated locations in the Western Indian Ocean. In the lab, we performed high-quality genomic DNA extraction from these muscle tissues using the sbeadex livestock kit. We then prepared ddRAD-seq libraries using EcoRI and Taq1a (New England Biolabs, Inc., Ipswich, MA, USA) and sent them for sequencing.

Timing and spatial scale	We collected the data between 2016-2017.
Data exclusions	No data where excluded from the analyses
Reproducibility	All analyses were repeated several times to confirm the robustness and accuracy of our results. Detailed documentation will accompany the code and data to facilitate their use by other researchers. We will provide the code and make the raw data available on a public data platform, ensuring transparency and reproducibility.
Randomization	We have different steps of randomization in our study:<br>First, the filtered SNP data were down-sampled 999 times to the lowest common number of SNPs (i.e., 4,479) found across all species.<br>Second, we applied a null model approach to test whether interspecific metrics differed from those expected at random. We first calculated the observed trait variability between separated locations of the Western Indian Ocean. Then, we randomized individuals between locations to obtain a distribution of 999 values of intraspecific trait variations. We calculated the standard effect size (SES) to determine whether to reject the null hypothesis (H0: the interspecific metric does not differ from that expected at random).<br>Similarly, to test whether intraspecific metric trait disparity (mtD) differed from that expected at random, we first calculated the observed trait variability between genera for a considered family. We then randomized species between genera of a family to obtain a distribution of 999 values of interspecific trait variations. We calculated the SES to determine whether to reject H0 (H0: the intraspecific mtD does not differ from that expected at random). This statistical framework was applied to each morphological trait ratio (n = 13).<br>To evaluate the congruence between intra- and interspecific morphological disparity, we performed a co-inertia analysis (CIA), with 99 randomization steps set up.
Blinding	We do not need to do Blinding during data aquistion because we want to targeted the most abundant species in tropical reef fishes species corresponding to a gradient of dispersal ability

Did the study involve field work? ☒ Yes ☐ No

Field work, collection and transport

Field conditions	Sampling at sea in the tropic by good condition
Location	Maldives, Mayotte Island, Mafia Island and Seychelles
Access & import/export	We thank the local authorities for issuing permits to collect samples and for help with field logistics in the Maldives, Mayotte (France island in Comorian archipelago), Seychelles and Mafia Island (Tanzania). Research permits number are: Maldives ((OTHR) 30-D/INDIV/2016/538), Mayotte (06/UTM/2016), Seychelles (A0157) and Tanzania (2017-242-NA-2017-87).
Disturbance	Sampling of adult reef fish species

Reporting for specific materials, systems and methods

We require information from authors about some types of materials, experimental systems and methods used in many studies. Here, indicate whether each material, system or method listed is relevant to your study. If you are not sure if a list item applies to your research, read the appropriate section before selecting a response.

Materials & experimental systems

n/a	Involved in the study	
☒	☐	Antibodies
☒	☐	Eukaryotic cell lines
☒	☐	Palaeontology and archaeology
☐	☒	Animals and other organisms
☒	☐	Clinical data
☒	☐	Dual use research of concern
☒	☐	Plants

Methods

n/a	Involved in the study	
☒	☐	ChIP-seq
☒	☐	Flow cytometry
☒	☐	MRI-based neuroimaging

Animals and other research organisms

Policy information about studies involving animals; ARRIVE guidelines recommended for reporting animal research, and Sex and Gender in Research

Laboratory animals	The study did not involve Laboratory animals.
Wild animals	We anesthetized the fish by placing them in a tank of water concentrated with clove oil. Once the fish were sedated, we placed them in a cooler at a temperature at 4°C.
Reporting on sex	We did not collect these data.
Field-collected samples	Samples were conserved in a freezer at -20°C in alcohol.
Ethics oversight	We applied Council Regulation (EU) No 1099/2009 of 24 September 2009 on the protection of animals at the time of killing.

Note that full information on the approval of the study protocol must also be provided in the manuscript.

Plants

Seed stocks	*Report on the source of all seed stocks or other plant material used. If applicable, state the seed stock centre and catalogue number. If plant specimens were collected from the field, describe the collection location, date and sampling procedures.*
Novel plant genotypes	*Describe the methods by which all novel plant genotypes were produced. This includes those generated by transgenic approaches, gene editing, chemical/radiation-based mutagenesis and hybridization. For transgenic lines, describe the transformation method, the number of independent lines analyzed and the generation upon which experiments were performed. For gene-edited lines, describe the editor used, the endogenous sequence targeted for editing, the targeting guide RNA sequence (if applicable) and how the editor was applied.*
Authentication	*Describe any authentication procedures for each seed stock used or novel genotype generated. Describe any experiments used to assess the effect of a mutation and, where applicable, how potential secondary effects (e.g. second site T-DNA insertions, mosiacism, off-target gene editing) were examined.*
